# Phanto-IDP: compact model for precise intrinsically disordered protein backbone generation and enhanced sampling

**DOI:** 10.1093/bib/bbad429

**Published:** 2023-11-28

**Authors:** Junjie Zhu, Zhengxin Li, Haowei Tong, Zhouyu Lu, Ningjie Zhang, Ting Wei, Hai-Feng Chen

**Affiliations:** State Key Laboratory of Microbial Metabolism, Joint International Research Laboratory of Metabolic & Developmental Sciences, Department of Bioinformatics and Biostatistics, National Experimental Teaching Center for Life Sciences and Biotechnology, School of Life Sciences and Biotechnology, Shanghai Jiao Tong University, Shanghai, 200240, China; State Key Laboratory of Microbial Metabolism, Joint International Research Laboratory of Metabolic & Developmental Sciences, Department of Bioinformatics and Biostatistics, National Experimental Teaching Center for Life Sciences and Biotechnology, School of Life Sciences and Biotechnology, Shanghai Jiao Tong University, Shanghai, 200240, China; State Key Laboratory of Microbial Metabolism, Joint International Research Laboratory of Metabolic & Developmental Sciences, Department of Bioinformatics and Biostatistics, National Experimental Teaching Center for Life Sciences and Biotechnology, School of Life Sciences and Biotechnology, Shanghai Jiao Tong University, Shanghai, 200240, China; State Key Laboratory of Microbial Metabolism, Joint International Research Laboratory of Metabolic & Developmental Sciences, Department of Bioinformatics and Biostatistics, National Experimental Teaching Center for Life Sciences and Biotechnology, School of Life Sciences and Biotechnology, Shanghai Jiao Tong University, Shanghai, 200240, China; State Key Laboratory of Microbial Metabolism, Joint International Research Laboratory of Metabolic & Developmental Sciences, Department of Bioinformatics and Biostatistics, National Experimental Teaching Center for Life Sciences and Biotechnology, School of Life Sciences and Biotechnology, Shanghai Jiao Tong University, Shanghai, 200240, China; State Key Laboratory of Microbial Metabolism, Joint International Research Laboratory of Metabolic & Developmental Sciences, Department of Bioinformatics and Biostatistics, National Experimental Teaching Center for Life Sciences and Biotechnology, School of Life Sciences and Biotechnology, Shanghai Jiao Tong University, Shanghai, 200240, China; State Key Laboratory of Microbial Metabolism, Joint International Research Laboratory of Metabolic & Developmental Sciences, Department of Bioinformatics and Biostatistics, National Experimental Teaching Center for Life Sciences and Biotechnology, School of Life Sciences and Biotechnology, Shanghai Jiao Tong University, Shanghai, 200240, China

**Keywords:** intrinsically disordered protein, protein backbone generation, molecular dynamic simulation, enhanced sampling, Phanto-IDP model

## Abstract

The biological function of proteins is determined not only by their static structures but also by the dynamic properties of their conformational ensembles. Numerous high-accuracy static structure prediction tools have been recently developed based on deep learning; however, there remains a lack of efficient and accurate methods for exploring protein dynamic conformations. Traditionally, studies concerning protein dynamics have relied on molecular dynamics (MD) simulations, which incur significant computational costs for all-atom precision and struggle to adequately sample conformational spaces with high energy barriers. To overcome these limitations, various enhanced sampling techniques have been developed to accelerate sampling in MD. Traditional enhanced sampling approaches like replica exchange molecular dynamics (REMD) and frontier expansion sampling (FEXS) often follow the MD simulation approach and still cost a lot of computational resources and time. Variational autoencoders (VAEs), as a classic deep generative model, are not restricted by potential energy landscapes and can explore conformational spaces more efficiently than traditional methods. However, VAEs often face challenges in generating reasonable conformations for complex proteins, especially intrinsically disordered proteins (IDPs), which limits their application as an enhanced sampling method. In this study, we presented a novel deep learning model (named Phanto-IDP) that utilizes a graph-based encoder to extract protein features and a transformer-based decoder combined with variational sampling to generate highly accurate protein backbones. Ten IDPs and four structured proteins were used to evaluate the sampling ability of Phanto-IDP. The results demonstrate that Phanto-IDP has high fidelity and diversity in the generated conformation ensembles, making it a suitable tool for enhancing the efficiency of MD simulation, generating broader protein conformational space and a continuous protein transition path.

## INTRODUCTION

In recent years, there has been significant progress in protein structure prediction. Deep learning models such as AlphaFold2 and ESMFold are already able to accurately predict the three-dimensional structure of a single protein based on its sequence [1, 2]. However, the biological function of a protein is not solely determined by a single three-dimensional structure, but rather, it also depends on the dynamic properties of its conformational ensemble [3]. Therefore, the characterization of protein conformational ensemble is indispensable for understanding its function, designing targeted drugs or any functionally related works [4, 5].

Experimental techniques, such as NMR, are used to probe the dynamic properties of biomolecules. However, these techniques often suffer from low resolution and are hardly applicable to intrinsically disordered proteins (IDPs) [6, 7]. Therefore, computational tools that can generate a collection of molecular conformations are urgently required as an alternative. One powerful strategy is molecular dynamic (MD) simulation, which is based on Newtonian mechanics that can sample conformations of biomolecules [8, 9]. MD simulation is frequently utilized in obtaining the energetically optimal regions and calculating the relative free energies between conformations [10, 11]. However, MD simulation is of high computational complexity and often struggles to cross kinetic barriers [12]. Therefore, various methods have been recently developed for accelerating MD sampling have been developed, collectively referred to as enhanced sampling.

Traditional enhanced sampling methods, such as replica exchange molecular dynamics (REMD), often follow the MD simulation approach and aim to accelerate the simulation process of crossing high energy barriers in the conformational space [13, 14]. Although these approaches do ensure more sufficient sampling, they still cost a lot of computational resources and time. On the other hand, in recent years, several deep learning–based enhanced sampling methods, such as the variational autoencoder (VAE), have been proposed [15, 16]. These methods usually train a generative model on existing MD simulation trajectories to efficiently generate novel protein conformations. However, the accuracy and rationality of the generated conformations by these models are often not satisfying, which undoubtedly limits the application of these methods in exploring protein dynamics [17, 18].

Considering the tremendous success of deep learning in the field of protein structure prediction in recent years, we believe that we can draw inspiration from these studies’ state-of-art decoders to improve the accuracy of existing generative models [19, 20]. In this study, we trained a generative model using full-atomic molecular dynamics simulation data of IDPs. The model functions akin to a phantoscope, being rather light-weighted and delving into the vast protein conformation space (named Phanto-IDP). We choose to train on IDP systems due to their high conformational diversity, which poeses challenges for traditional experimental or computational methods [21]. We represented the target protein conformation in the form of graphs and constructed a modified graph variational autoencoder. Inspired by cutting-edge protein structure prediction models, we used transformer as the decoder part of the model, enabling the model to output Cartesian coordinates directly. Notably, the average root-mean-square deviation (RMSD) between the backbone reconstructed by our model and the input conformation in most tested protein systems were less than 1 Å (e.g., 0.885 Å in PaaA2, a 71-residue IDP), indicating that our model is accurate in predicting protein structure. Furthermore, our model exhibits impressive speed, generating 50 000 conformations within 1 min, with many being previously unseen. By customizing the sampling distribution in the latent space, we could use the model to sample a conformational ensemble to describe possible transition paths in detail. We also proved that the generated ensemble conforms to Boltzmann distribution; thus, the generated conformations could be directly used for calculating relative free energies or downstream applications such as drug screening.

## RESULTS

### Overview

**Figure 1:**
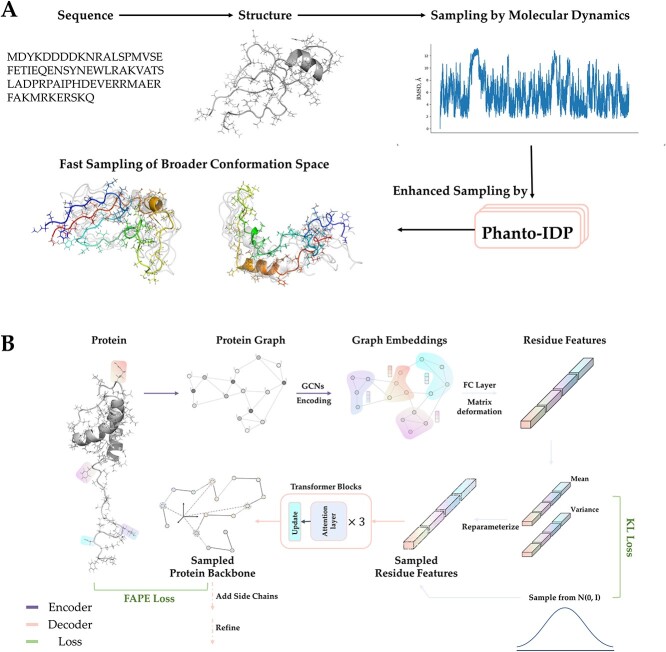
Overview of Phanto-IDP. (**A**) The way Phanto-IDP functions in sampling conformation space for a specific protein. (**B**) Architecture of Phanto-IDP.

Given a conformation from MD trajectory, Phanto-IDP first converts the backbone atom features into a protein graph and uses the graph convolutional network (GCN) to learn the atom embeddings. Then, a non-linear fully connected layer is applied to the embedded graph for feature dimension reduction. The next step involves transforming the atom embedding features into residue graphs, which are subsequently fed into the variational layer. Finally, transformer blocks are utilized to obtain the backbone atom coordinates (shown in Figure 1). Please refer to Materials and Methods for more details.

To assess the effectiveness of our model, we designed several tasks to evaluate the performance of Phanto-IDP. Firstly, we assessed the model’s capability to reconstruct IDP conformations, which determined the model’s ability to extract information from graph representations and to accurately capture crucial features. Satisfying reconstruction performance is a prerequisite of generative sampling, as it signifies the model’s proficiency in capturing structure variation at sufficient resolution. Next, we analyzed the diversity and quality of generated conformations and visualized the conformation ensembles through principal component analysis (PCA) dimensionality reduction and clustering. We further explored the potential of the model for enhanced sampling. By training and generating on short MD trajectories, we investigated the similarities and differences between the conformation ensembles generated by the model and those obtained from replica exchange molecular dynamics (REMD). Additionally, we utilized Phanto-IDP to generate interpolations between two extremely different conformations selected from original MD trajectories. These tests served as evidence that our approach could be effectively used for enhanced sampling.

### Reconstruction of IDP conformations

A fundamental request for a generative model is its capacity to accurately embed and reconstruct data. Precise reconstruction demonstrates the capability of a model to effectively capture structural features. This ability is crucial for generating unseen conformations through latent space sampling.

**Figure 2:**
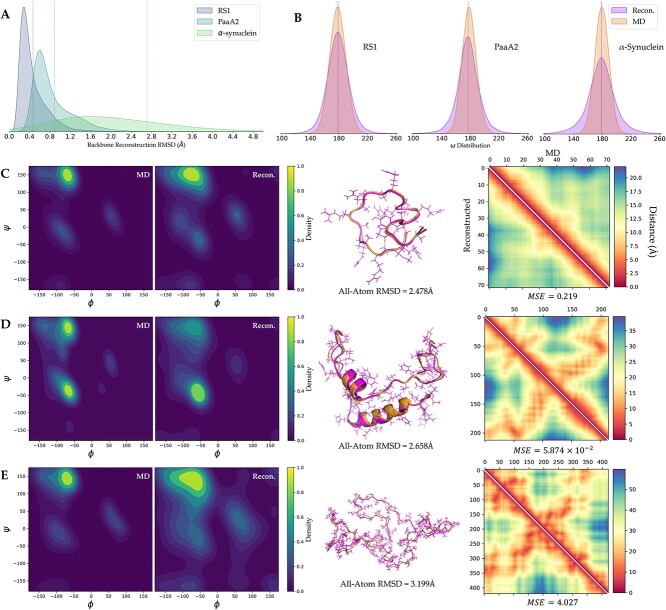
Evaluation of conformation reconstruction on IDP systems. (**A**) Pairwise RMSD distribution between unrefined reconstructed backbone and ground truth (from MD trajectories), 12.5 k conformations from test set are involved in this plot. (**B**) Distribution of $\omega$ dihedrals from refined conformations. (**C**–**E**) Ramachandran plot, ground truth structure and reconstructed structure (with side chain shown) of (C) RS1, (D) PaaA2 and (E) α-synuclein. Average contact maps of MD (in upper triangles) and reconstructed ensembles (in lower triangles) are displayed besides the structures. Each Ramachandran plot involves 20 k dihedrals.

We trained and evaluated the model using 50% of the conformations from MD trajectories as the training set, 25% as the validation set and 25% as the test set. On the test set, which consists of 12 500 conformations that were not encountered during the model training process, we assessed the quality of conformation reconstruction using RMSD and dihedral angle distributions as indexes (shown in Figure 2).

First, we calculated the pairwise RMSD between the backbone of all input conformations and their corresponding reconstructed conformations (Figure 2A). The average reconstruction RMSD for the RS1, PaaA2 and α-synuclein systems was 0.511, 0.885 and 2.714 Å, respectively. These reconstruction errors could be considered as slight. Additionally, we computed 20 k dihedral angles, respectively, for the input and reconstructed conformation ensembles. The results show that the distribution of reconstructed conformations closely matches the $\omega$ dihedral angle distribution of the MD conformation ensemble (Figure 2B). We further calculated Jensen–Shannon (JS) divergence between the reconstructed ensemble and the MD-derived $\omega$ angles. The low divergence (0.035 in RS1, 0.052 in PaaA2 and 0.102 for α-synuclein) indicated high similarity between the reconstructed omega angles and the original ones across the three systems.

We also calculated the $\varphi -\psi$ distributions of the reconstructed conformation ensembles and compared them with MD trajectories (Figure 2C–E), noticing that $\varphi -\psi$ distributions of the reconstructed conformation ensembles were quite similar to the MD distributions. We randomly selected a set of original conformations and reconstructed conformations for each of the three IDP systems and performed alignment and visualization using PyMOL (https://pymol.org/2/). The all-atom RMSDs of these three sets of conformations were slightly higher than the backbone RMSDs, indicating that the refinement process might not fully reconstruct the side-chain distributions of the corresponding conformations. However, refinement did not affect backbone distribution severely (Figure S1), and the reconstructed backbone was considered relatively ideal. This could also be inferred from the similarity analysis between the average distance maps of MD and the reconstruction ensembles (Figure 2C–E). The MSE of the average distance matrices among the three test systems was relatively small. In the PaaA2 system, $\mathrm{MSE}=5.874\times{10}^{-2}$, which we attributed to its high degree of structural organization and low difficulty in learning ordered features. When analyzing the reconstruction performance of the model, we found that due to the limited motion of structured regions in the MD trajectories and their relatively fixed features, the accuracy of the model in reconstructing these regions was much higher than that of disordered regions (Figures S2–S5). This phenomenon is consistent with the low confidence often observed in disordered regions in previous protein structure prediction or generation tasks, indicating the necessity of our model for reconstructing and characterizing the diversity of disordered regions [22].

### Generation of unseen conformations

**Figure 3:**
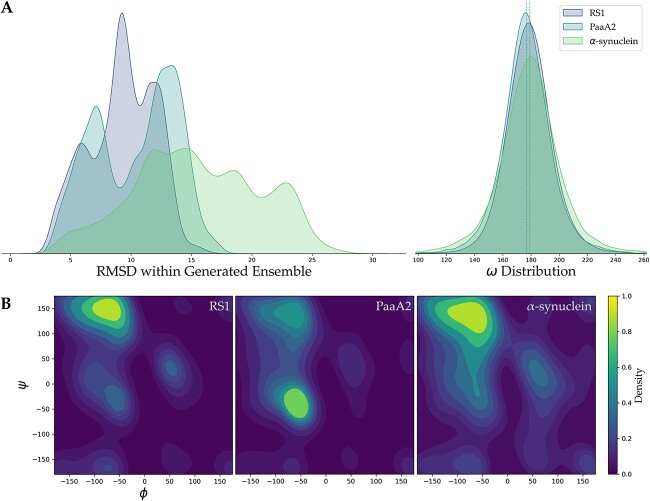
Evaluation of generated conformation ensembles. (**A**) One-against-all RMSD and distributions within three generated ensembles, respectively. (**B**) Ramachandran plot of three generated IDP ensembles. Each Ramachandran plot involves 20 k dihedrals.

For the generation task, we first employed evaluation metrics similar to those utilized in reconstruction task, calculating RMSD and distribution of the three main-chain dihedral angles. Here, we computed one-against-all RMSD within the generated conformation ensembles, which helped us observe the diversity of the generated conformations (Figure 3A). The results show that RMSD within the three generation sets distribute quite broad, including pairs of conformations with RMSD greater than 30 Å in the generation of α-synuclein. This indicated that the model trained on MD trajectories could capture the differences in conformations within the latent space and obtain significantly different conformations by sampling different regions in the latent space. Meanwhile, the $\omega$ angle distribution was well preserved in the generated ensembles (Figure 3A), while the $\varphi -\psi$ distribution was slightly broader than that of MD and reconstruction ensembles (Figure 3B). The dihedral distribution in the Ramachandran plot still remained mostly in the reasonable region, indicating that the generated conformations could maintain the rationality of the main-chain structures well.

**Figure 4:**
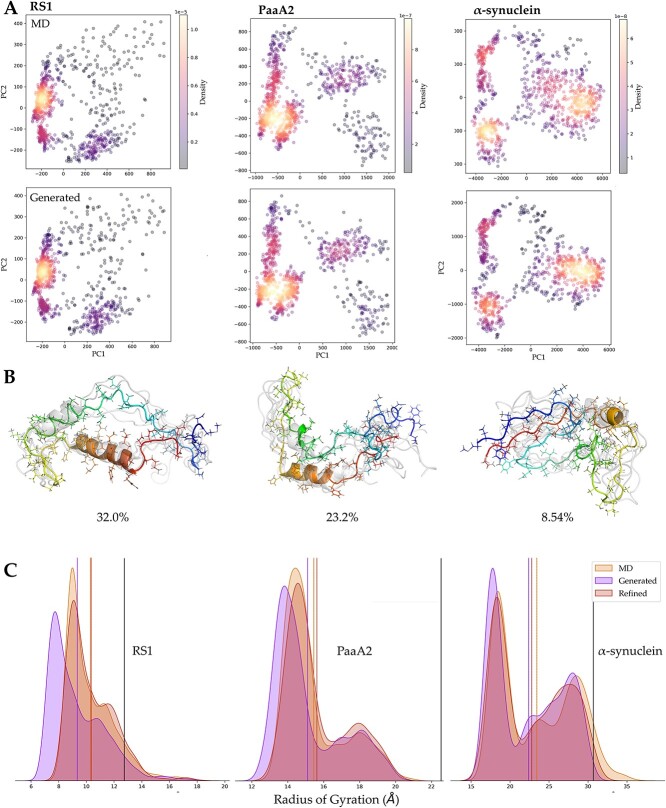
Diversity of generated conformation ensembles. (**A**) PCA visualization of MD trajectories (upper) and generated conformation ensembles (below). (**B**) Top three cluster centroids of generated PaaA2 conformation ensembles. (**C**) Distribution and average value (solid lines) of radius of gyration calculated from directly generated, refined and MD conformation ensembles (black line denotes experimental data).

PCA was utilized on the generated conformations and MD trajectories simultaneously to explore the relationship between the global distribution of two ensembles (Figure 4). We calculated the distance maps for each conformation and flattened them as high-dimensional feature vectors, which were then subjected to PCA for dimensionality reduction. The dimensionality-reduced generated conformation ensembles were found to be consistent with MD trajectories in their distribution shape. It’s also observed that the generated ensemble contained samples not observed in the MD trajectories, indicating that the model could globally fit the converged Boltzmann distribution of the MD trajectories and could be used to generate previously unseen conformations. These conformations might correspond to some saddle points on the potential energy surface that are difficult to sample using traditional MD methods.

We further clustered the generated conformations based on RMSD and visualized the three most predominant cluster centers (Figure 4A, Figure S6). For each cluster, we displayed five conformations including the centroid (in rainbow color); it was evident that there were significant differences among these cluster centers, indicating high diversity in the generated conformation ensembles. For PaaA2, conformations in the first cluster mostly had two clear helices, the second contained conformations that owned shorter helices with more disorder and the third cluster included even more disordered conformations with only one helix. Additionally, Figure 4B displayed the radius of gyration (Rg) distributions of the MD trajectories, directly generated and refined conformation ensembles for the three systems. It could be observed that Rg distributions of the directly generated structures were slightly lower than the actual MD distributions, which was mainly resulted from lack of side chains. As a proof, Rg distributions of the refined structures were in good agreement with the MD distributions. In summary, our model is capable of capturing the actual conformational diversity and global properties observed in MD trajectories during the generation process while preserving the underlying Boltzmann distribution.

### Comparison with previous methods

We compared Phanto-IDP with MD and previously developed deep learning models [16, 17]. The models’ ability to reconstruct individual conformation in the trajectory was evaluated using average RMSD, which measured the level of fidelity in capturing conformational features and diversity at the conformational level. We also calculated the JS divergence between Rg of conformation ensembles generated by these deep learning methods and MD trajectories to assess whether the models could accurately reproduce the distribution properties of the MD trajectory and capture the global features of conformational ensembles. Finally, we recorded the speed of generating conformations using the converged model on Tesla V100. We generated 50 000 conformations with each model, which is comparable to the number of conformations extracted from a 1 μs MD trajectory.

The results show that our model has the lowest reconstruction RMSD and JS divergence on all tested systems, indicating that Phanto-IDP is a more precise model compared to the previous deep learning models (Table 1, Table S1). From the record of generation time for 50 000 conformations, we found that Phanto-IDP did not consume as much time as AE or VAE when the target protein got bigger, which complied with the designed model architecture. Phanto-IDP has a fixed number of learnable parameters (a total of 142 k), and the computational complexity for different proteins should be linearly related to the number of input atoms. Since the features provided only include backbone atoms, the speed of model reconstruction and generation should be linearly related to the sequence length of input protein. In contrast, for traditional fully connected networks, an increase in the length of the input protein significantly increases the number of model parameters and computational complexity. Therefore, Phanto-IDP is designed to operate faster than traditional methods on larger systems. For the system with 140 residues, α-synuclein, our model could generate a comparable number and diversity of conformations to the MD trajectory in approximately 50 s, which was extremely fast compared to MD and previous models. In practical use, we trained Phanto-IDP for 400 epochs to ensure sufficient convergence (although we observe that the loss function converges in around 200 epochs). This training process took 30.91 h on α-synuclein. In other words, we could complete training process and generate any number of conformations within 2 days, which was significantly faster compared to the 270 h required for MD to obtain a 1 μs equilibrium system.

**Table 1:** Comparison of Phanto-IDP against traditional methods.

Protein	Method	Avg. RMSD, Å	JS divergence of Rg	Speed
RS1	MD (1 μs)			245.14 h
	AE	9.892	0.121	**13.61 s**
	VAE	7.115	0.905	64.26 s
	Phanto-IDP	**0.511**	**0.036**	28.22 s
PaaA2	MD (1 μs)			251.75 h
	AE	9.417	0.514	49.72 s
	VAE	8.341	1.823	87.03 s
	Phanto-IDP	**0.885**	**0.093**	**34.54 s**
α-Synuclein	MD (1 μs)			270.50 h
	AE	12.433	1.223	79.09 s
	VAE	10.417	1.892	111.40 s
	Phanto-IDP	**2.714**	**0.105**	**50.60 s**

Bold values represents best performance among all methods.

### Enhanced sampling

Here, we proposed two scenarios suitable for using Phanto-IDP as an enhanced sampling technique. The first scenario involved using the model to interpolate the conformational space on existing large-scale trajectories. Effective and continuous conformational space interpolation could help us characterize more meticulously the dynamic properties of the target protein, especially how the protein transits from one macrostate to another. This is crucial for understanding proteins, especially the physiological properties of IDPs. The second scenario involved training on short MD trajectories and generating unseen conformations that might be rare even in long trajectories or REMD. These novel conformations could either be used for further MD simulations or help traditional MD overcome barriers that are difficult to cross.

**Figure 5:**
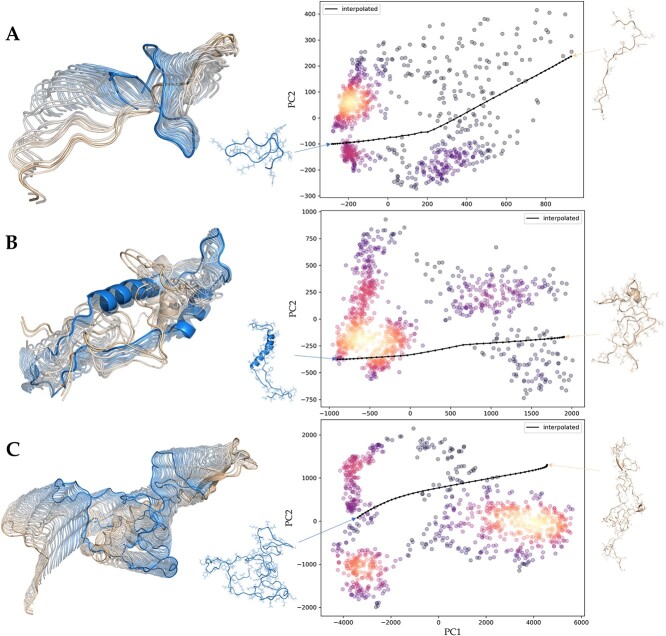
Interpolation between two states on three IDP systems. The structure on the left denotes starting point of conformation interpolation, and the structure on the right denotes the end point. The colors of the output conformations correspond to their positions in the latent space, illustrating the gradual change in protein structure along the interpolation path. (**A**) RS1. (**B**) PaaA2. (**C**) α-Synuclein.

We demonstrated the success of Phanto-IDP in these two enhanced sampling tasks through two tests. For the first task, we selected two rather different conformations from the three target IDP systems and used Phanto-IDP to linearly interpolate these two conformations in the latent space of the model. A collection of conformations on this path was obtained through the decoder and was visualized in Figure 5 (left). The interpolated conformations performed a continuous transition, indicating the potential pathway of how the target protein transited from one discrete conformation to another. We mapped the interpolated conformation collection to the PCA space constructed in the previous generation task and observed their spatial distribution relationship (Figure 5, right). The linear interpolation in the latent space of the model exhibited an obvious nonlinear path in the PCA space, proving that the latent space captured complex nonlinear features of target systems. From the PCA result, we found that the interpolated path might well cross areas that was not sampled in MD, meaning that the generated ensemble is of diversity beyond the original trajectories.

For the second task, we trained our model on a relatively short MD trajectory and compared the properties of the generated ensemble with those of an REMD trajectory.

**Figure 6:**
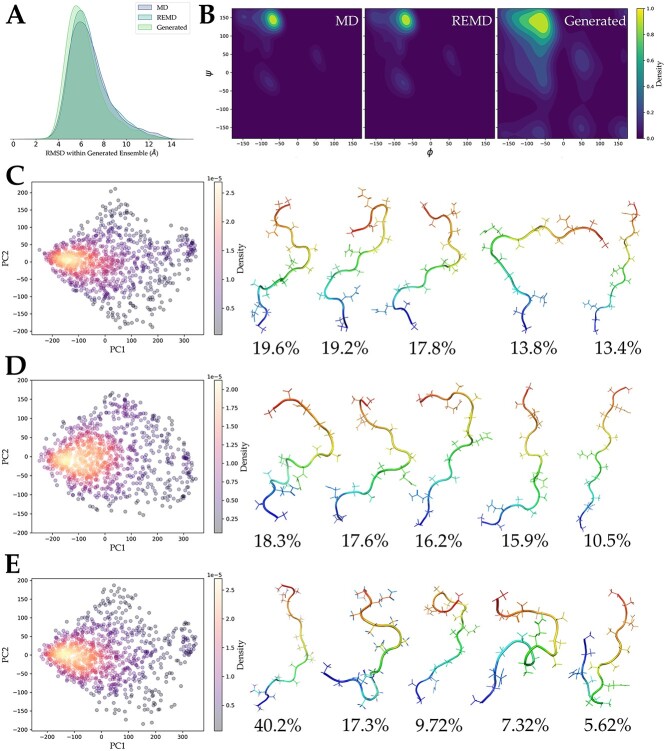
Performance of model generation based on short MD trajectory. (**A**) One-against-all RMSD within generated ensemble compared to original MD and REMD. (**B**) Ramachandran plots of generated ensemble, conventional MD and REMD. PCA and clustering result: (**C**) Conventional MD. (**D**) REMD. (**E**) Generated ensemble.

We computed the one-against-all RMSD and $\varphi -\psi$ distributions within the generated conformational ensemble and compared them with trajectories from traditional MD and REMD. We found that the RMSD distributions of the three methods were essentially the same (Figure 6A), indicating a similar level of conformational diversity. However, from the Ramachandran plot, it was observed that the generated conformational ensemble sampled a wider range of dihedral angles, indicating that the model likely sampled conformations unseen in both conventional MD and REMD (Figure 6B). To validate this point, we further performed PCA dimensionality reduction on the generated conformational ensemble and two MD trajectories, followed by clustering analysis based on RMSD (Figure 6C–E). The sampling ranges in PCA space of the generated ensemble, conventional MD and REMD were not significantly different. However, from the clustering results, the conformations sampled by MD were mostly in a relatively disordered state, possibly due to the short simulation failing to adequately sample folded protein conformations. Nevertheless, in the generated ensemble from Phanto-IDP trained on short simulation, we observed 5.62% of structures in a folded state, and these structures already showed a tendency to form helices. Although the proportion of folded conformations generated by our model was still lower compared to the 18.3% sampled in REMD, considering that such folded conformations were not present in the training set, the model was proved to be able to cross energy barriers and sample novel conformations that were difficult to explore in conventional MD.

## DISCUSSION

We utilize a compact deep learning model, Phanto-IDP, to learn from multiple trajectories of IDPs and structured proteins, demonstrating its ability to generate reasonable and diverse conformations and perform enhanced sampling tasks. The model is trained on all-atom molecular dynamics simulation data, utilizing graph networks to extract protein features and efficiently generating conformational ensembles unseen in the trajectories through a variational sampling technique and a transformer-based decoder. As there’s no physics-based iterative sampling process, the model generates conformations much faster compared to traditional MD or enhanced sampling methods. Despite this speed advantage, the generated conformation ensembles retain the global distribution properties observed in the trajectories, indicating that the generated ensembles conform to the Boltzmann distribution as convergent trajectories.

In the initial stages of our research, we employed a multilayer perceptron (MLP) as the decoder in our model. However, the generated conformations exhibited numerous clashes and inconsistencies. To address this challenge, we transited to a transformer-based decoder architecture, resulting in a noticeable improvement in reconstructing conformations. Nevertheless, we still faced challenges when it came to generating novel conformations that met our expectations. Assigning a high weight to the KL loss could be a solution, but it also led to problems like posterior collapse and hinder overall training progress, a gradual increase in the weight of the KL loss during training. This approach struck the right balance between reconstruction and generation performance, ultimately shaping the training scheme of Phanto-IDP. The training process for all three versions of Phanto-IDP is shown in Figure S7.

Compared to previous deep learning–based all-atom enhanced sampling methods, Phanto-IDP is SE (3) equivariant and can more accurately capture protein backbone and global features. The performance of our model in enhanced sampling is comparable to traditional methods like REMD, suggesting that Phanto-IDP is an efficient enhanced sampling tool. In addition, we compared with the general protein conformation generation models, such as FoldingDiff and EigenFold, to forecast the conformational ensembles of our selected IDP systems [20, 23]. It’s important to note that these general models are typically trained on datasets like PDB or CATH, which contain limited information regarding disordered regions. These models exhibited worse performance than that of Phanto-IDP, as illustrated in Figure S8. Furthermore, Phanto-IDP is characterized by its compact size, featuring only 142 k parameters. Numerous studies have confirmed that augmenting the size of transformer-based models often leads to enhanced performance [2, 24]. Therefore, incorporating more sophisticated and larger components into Phanto-IDP might yield improved generation capabilities.

Phanto-IDP cannot be directly transferred from one system to another without additional training. Though the model size does not change with the size of the target protein, direct transfer might lead to severe misjudgment of structure details. However, *ab initio* training does not take a long time as MD simulation consumes. In our cases, it takes no longer than 2 days on proteins with under 200 residues using one Tesla V100.

In this study, our model performs satisfying in conformation reconstruction, generation and interpolation (Figures S2–S5, S9–S15) on 13 protein systems, thus demonstrating its robustness. However, since we only train the model on MD trajectories, it is challenging to achieve better performance in replicating experimental observations. In terms of Rg, the model-generated ensemble reaches a level very close to the MD trajectories, but there is still a significant deviation between the mean Rg values of the simulated and generated ensembles and the experimental values. Regarding J-coupling, we obtain six IDP systems with experimental values among the 10 tested ones. In the case of RS1 and drkN SH3 domain, J-coupling values of the generated ensemble are closer to the experimental values compared to MD simulations. However, the generated results are not as good as MD simulations for the other four systems (Figure S16). Considering that J-coupling calculations should only depend on dihedral angles, we presume that the instability in J-coupling performance is due to the fact that the dihedral angle distributions in the generated conformation ensemble are consistently broader than those within MD simulations. In reality, broader distribution of dihedral angles in experimental structures might result in better generated results than MD simulation, or conversely, it might lead to worse result. In this regard, we believe that in the next step, we should involve pre-training the model on the PDB database, enabling it to capture the diversity present in experimental structures, followed by fine-tuning with MD simulation trajectories specific to different systems [25]. Additionally, more accurate force fields and solvent models should be employed for simulations [26, 27].

The model effectively preserved the Boltzmann distribution observed in convergent trajectories; however, obtaining such convergent trajectories in practice remains a challenging task [21]. Training the model on non-equilibrium systems might lead to the generated global distribution not capturing real kinetics. In other words, the enhanced sampling performed by Phanto-IDP might fail to produce conformation ensembles with realistic dynamic properties when applied to input trajectories that are non-equilibrium. To address this issue, on the one hand, we can restore the known system’s kinetics through simulation-based resampling [28]. On the other hand, we can improve the generation approach of Phanto-IDP by incorporating temporal autocorrelation constraints or constructing hierarchical models to better capture dynamic properties [29, 30]. Another alternative worth considering is the use of the Boltzmann generator, which could offer capability to capture dynamic properties. However, their use of standard flow models places high demands on network design, and there have been few developments in this regard in recent years [31]. Recently, several diffusion models have emerged, employing energy-based training methods to generate molecular conformations [25, 32]. This approach, characterized by its data-free strategy, trains the model to effectively capture the equilibrium distribution. Such an approach holds the promise of enhancing Phanto-IDP’s performance in non-equilibrium systems.

In terms of network design, the architecture of Phanto-IDP is relatively simple. This is because the model needs to be trained on the target system, and overly complex designs might lead to unacceptable training time and memory usage. From a practical perspective, it is preferable to initially pretrain the network on multiple systems and fine-tune the network parameters for specific applications [33]. Although the current Phanto-IDP can accept systems with different topologies during training, for this study, we chose not to implement this approach due to concerns about the instability of cross-system learning. In the next step, we plan to explore the method of cross-system training and transform Phanto-IDP into a pretrained conditional generative model. Several studies have attempted to use diffusion models to address this task, but due to restriction of complexity, these studies often work on coarse-grained structural data. We believe that using the lightweight network architecture of Phanto-IDP to build a diffusion model has the potential to generate higher-precision structures.

Using Phanto-IDP for enhanced sampling offers advantages in terms of both accuracy and time cost compared to previous methods. However, a challenge arises from the model’s strong dependence on the training data. For example, training the model on a trajectory of a protein in its apo state, it remains challenging to generate possible conformations of the protein in its holo state. This problem could potentially be addressed through cross-system training, as it allows the model to receive more protein topology information during training, thereby assisting the model to generate more diverse conformations. In the next phase, we will focus on improving the robustness and diversity of Phanto-IDP generation by overcoming the limitations of limited information from MD trajectories through cross-system pretraining. The code of Phanto-IDP is deposited at https://github.com/HFChenLab/PhantoIDP.

## MATERIALS AND METHODS

### Dataset

#### MD simulations

In our previous works on MD simulation, a series of converged simulations on IDP systems were obtained [34, 35]. Here we chose 10 IDPs and four ordered proteins of length less than 200 amino acids and properties as our main target (Table 2). We simulated the proteins with force field ESFF1 and solvent model OPC3 for 1 μs and extracted 50 000 conformations at 0.02 ns interval for model training and evaluation [36, 37]. We comprehensively evaluate the performance of our model in reconstructing and generating protein conformations, including metrics like RMSD, $\mathrm{\varphi} -\mathrm{\psi}$ distribution, $\mathrm{\omega}$ angle distribution, contact map, Rg distribution, PCA and clustering results. The trajectories involved in this study were proved to converge in the previous research [27].

**Table 2:** Protein systems and corresponding properties involved in this study

Protein	Length, aa	Experimental Rg	Simulation condition
Intrinsically disordered proteins			
RS1	24	12.62 [1, 2]	ESFF1/OPC3 1000 ns [3]
PaaA2	71	22.4 [4]	
α-Synuclein	140	31.5 [5, 6]	
Histain5	24	13.8 [7, 8]	
Aβ40	40	12 [9, 10]	
Aβ42	42	12.4 [10, 11]	
drkN SH3 domain	59	16.7 [12]	
ACTR	71	25 [13]	
R17	100	22.9 [14]	
p15PAF	110	28.1 [15]	
Folded proteins			
CspTm	66	11.07 [16]	ESFF1/OPC3 1000 ns [3]
Ubiquitin	76	11.63 [17]	
SPR17	118	118 [6]	
AAQAA3	15	–	ESFF1/OPC3 500 ns
			REMD 1200 ns [18]

#### Data split

For the 10 IDPs and three structured protein systems, to train our model to fully capture the diversity of their conformation ensemble, we shuffled the trajectories from MD simulation and split the dataset into the training set, evaluation set and test set according to a preset proportion. The similarity between the training set and the test set is evaluated through RMSD and PCA analysis as shown in Figure S17. Conformations from the two sets obey similar distribution, while they typically own an RMSD of over 2 Å, indicating that they are different in local characters.

For short MD simulation on small peptide AAQAA3, we use 80% of the trajectory for training as we expect that the model capture most of the dynamics feature.

#### Graph representation

We preserve backbone atoms (N, CA, C) for each input conformation and construct a crystal graph at the atomic level. Let $\mathcal{G}=\left(\mathcal{V},\mathcal{E}\right)$ be a graph, where $\mathcal{V}=\left({v}_1,\dots, {v}_m\right)\in{\mathbb{R}}^{m\times \mathrm{\chi}}$ denotes the set of vertices with $\mathrm{\chi}$-dimensional input features and $\mathcal{E}=\left({e}_{11},{e}_{12},\dots, {e}_{mk}\right)\in{\mathbb{R}}^{m\times k\times \mathrm{\omega}}$ represents the set of edges with $\mathrm{\omega}$-dimensional input features. Here, $m$ is equal to $3\times$ residue number of the input conformation, as we only retain three backbone atoms for each residue. When calculating the adjacency matrix, we use the K-Nearest Neighbors (KNN) algorithm which connects only $k$ nodes ($k=30$ by default) that are nearest in physical distance for each node.

For node feature, we treat N, CA, C in the 20 standard residue types separately, which yields 60 atom types, so that the node feature is constructed as a one hot vector with $\mathrm{\omega} =60$. For edge feature, we combine three SE (3) equivariant characters that can define both global and local structures well:

(i) Physical distance between two atoms: We calculate the Cartesian distance between each two atoms connected in graph and generate a vector representation using gaussian basis expansion. We sampled 16 means uniformly from [0, 20] for Gaussian basis in this work.
(ii) Relative atom position in local frames: We treat every atom as center of a local reference frame $\overrightarrow{n_x},\overrightarrow{n_y},\overrightarrow{n_z}$, which is calculated with two bonded atoms A, C and the center atom B (Equations (1)–(3)).

(1) \begin{equation*} {\displaystyle \begin{array}{c}\overrightarrow{n_x}=\frac{\overrightarrow{\mathrm{BC}}\times \overrightarrow{\mathrm{AB}}}{\left|\overrightarrow{\mathrm{BC}}\times \overrightarrow{\mathrm{AB}}\right|}\end{array}} \end{equation*}

(2) \begin{equation*} {\displaystyle \begin{array}{c}\overrightarrow{n_z}=\frac{\overrightarrow{\mathrm{AB}}\times \overrightarrow{\mathrm{BC}}}{\left|\overrightarrow{\mathrm{AB}}\times \overrightarrow{\mathrm{BC}}\right|}\end{array}} \end{equation*}

(3) \begin{equation*} {\displaystyle \begin{array}{c}\overrightarrow{n_y}=\overrightarrow{n_z}\times \overrightarrow{n_x}\end{array}} \end{equation*}

In any edge ${e}_{ij}$, we project atom $j\ (i)$ onto the frame of atom $i\ (j)$ so that we get two relative atom positions as an additional feature in adjacent matrix. Calculation of local frames and projections is done with the mylddt toolset (https://github.com/gjoni/mylddt).

(iii) Bond types: The final part of edge feature is binary, where 1 represents that two atoms are bonded in actual structure and 0 for otherwise.

#### Model architecture

Phanto-IDP is designed to be a generative graph neural network whose encoder is composed of three graph convolutional layers and decoder consists of three transformer blocks. For generation task, we added a variational layer between the encoder and decoder, the same as that in a variational autoencoder. In this section we’ll introduce the three parts of our model separately.

#### Encoder

As described above, we processed the input protein conformations into crystal graphs. Here, we employ one hot vectors for vertices features, so an embedding layer is utilized for transforming the input discrete features into a 64-dimensional continuous vector as input of encoder.

We used graph convolutional network (GCN) as encoder because it can effectively incorporate edge information and information from neighboring nodes into the current node [38]. The message passing rule in the ${k}^{th}$ layer of GCN is defined as follows:


(4) \begin{equation*} {\displaystyle \begin{array}{c}\;{h}_{i,j}^{(k)}={v}_i^{(k)}\oplus{e}_{i,j}^{(k)}\oplus{v}_j^{(k)}\end{array}} \end{equation*}

(5) \begin{equation*} {\displaystyle \begin{array}{c}{v}_i^{k+1}={v}_i^k+{\sum}_{j\in{\mathcal{N}}_{\mathcal{i}}}\sigma \left({h}_{i,j}^{(k)}{W}_g^{(k)}+{b}_g^{(k)}\right)\odot R\mathrm{eLU}\left({h}_{i,j}^{(k)}{W}_c^{(k)}+{b}_c^{(k)}\right)\end{array}} \end{equation*}

where ${h}_{i,j}^{(k)}$ denotes the hidden representation of ${v}_i^k$ with neighbor information concatenated (Equation (4)). The hidden representation then undergoes an edge-gating mechanism (Equation (5)) to incorporate different interaction strengths among neighbors, where $\sigma$ denotes a sigmoid function, ${W}_g^{(k)},{b}_g^{(k)}$ are gate weight matrix and bias separately and ${W}_c^{(k)},{b}_c^{(k)}$ are convolutional weight matrix and bias, respectively.

#### Variational inference module

To ensure stronger generalization and generation ability, we added a variational inference module between the encoder and decoder, referring to the variational autoencoder (VAE) described by Kingma and Welling [39]. This module first transforms the node feature obtained from encoder to a low-dimensional latent space through a fully connected (FC) layer. Then two FC layers are utilized for calculation of mean and logarithmic variance matrices in the latent space. With the trick of reparameterization (Equation (6)), we sample from the neighboring area of the encoding feature in the latent space but not just the exact point.


(6) \begin{equation*} {\displaystyle \begin{array}{c}z=\mu +\sigma \ast \epsilon \ast \mathrm{T}\end{array}} \end{equation*}

where $\mu$ is the mean matrix and $\sigma$ is the variance matrix. $\epsilon$ is a random variable sampled from a standard normal distribution and $T$ denoting temperature determines the actual scale of sampling. $T$ is set as 0.02 in default for generation tasks in this study, as we find that under this temperature the generated conformations are of high rationality while capturing enough diversity.

With variational inference module, our model can learn not only the point information corresponding to the input conformation but also the distribution information in the whole latent space. During training, we use KL divergence to limit the neighboring area of the sampled distribution to be close to the standard normal distribution. In this way, the well-trained model is able to generate a large number of unseen protein conformations with seeds from normal distribution.

#### Decoder

The decoder receives features sampled from the variational inference module and further transform the latent features into Cartesian coordinates of corresponding protein conformation. Here, idpGAN is consulted to construct our decoder that consists of three transformer blocks; each contains a self-attention layer and an update module (Figure 1) [21].

### Loss function and training strategy

As we build a hybrid generative model based on the principle of VAE, the loss function used in training model is naturally composed of two parts (Equation (7)), reconstruction loss and Kullback–Leibler (KL) loss.


(7) \begin{equation*} {\displaystyle \begin{array}{c}\mathcal{L}={\mathcal{w}}_{\mathrm{Recon}}{\mathcal{L}}_{\mathrm{Recon}}+{\mathcal{w}}_{\mathrm{KL}}{\mathcal{L}}_{\mathrm{KL}}\end{array}} \end{equation*}

### Reconstruction loss

Here, to construct an SE (3) equivariant model, we utilize the frame-aligned point error (FAPE) as the reconstruction loss to ensure that the output coordinates are equivariant to the input ones in rotation and translation [1, 40]. We first transform the output backbone atom coordinates into corresponding residual local frames using the Gram–Schmidt process (Equations (1)–(3)), then FAPE loss on backbone is calculated according to AlphaFold2 (Equation (8)), which can be written as followed.


(8) \begin{equation*} {\displaystyle \begin{array}{c}{\mathcal{L}}_{\mathrm{Recon}}=\frac{1}{N_{\mathrm{frames}}{N}_{\mathrm{atoms}}}{\sum}_{\mathrm{i},\mathrm{j}}\left\Vert{T}_{i\left(\mathrm{output}\right)}^{-1}\circ{\overrightarrow{x}}_{j\left(\mathrm{output}\right)}-{T}_{i(gt)}^{-1}\circ{\overrightarrow{x}}_{j(gt)}\right\Vert \end{array}} \end{equation*}

where ${T}_i$ denotes local frame of the $i- th$ amino acid and ${\overrightarrow{\mathrm{x}}}_j$ is coordinate vector of the $j- th$ backbone atom. Here, $\mathrm{output}$ represents the conformations generated by our model and $gt$ represents the ground truth in model training, in other words, the conformations from MD simulation.

### KL loss

KL loss is a regularization term that encourages the encoder to produce latent variables that are close to a standard normal distribution. This term measures the difference between the learned distribution of the latent variables and the target distribution, which is a standard normal distribution in this work (Equation (9)). The KL loss term helps to constrain the latent space to be more interpretable and easier to manipulate, which can improve the quality and interpretability of the generated data.


(9) \begin{equation*} {\displaystyle \begin{array}{c}{\mathcal{L}}_{\mathcal{KL}}=\mathbb{E}\left[\frac{1+\log \left(\sigma \right)-\mu -\sigma }{2}\right]\end{array}} \end{equation*}

where $\mu$ and $\sigma$ are the mean and variance matrix in Equation (3), respectively.

### Weight setting

In training the model, we find that it’s much easier to optimize KL loss than to optimize reconstruction loss. However, excessive optimization of KL loss results in loss of information from encoder, which is referred to as posterior collapse [41]. Posterior collapse can severely affect the reconstruction performance of the model, so we dynamically set the weight of KL loss to prevent such a problem [42]. Detailed weight setting is recorded in Table 3 as follows.

**Table 3:** Weight setting of KL loss and FAPE loss, respectively

Weight	Value _Iterations_
${\mathcal{w}}_{\mathrm{Recon}}$	${10.0}_{0.3M}$ , ${1.0}_{0.1M}$
${\mathcal{w}}_{\mathrm{KL}}$	$1.0\times{10}_{50K}^{-4}$ , $5.0\times{10}_{50K}^{-4}$, $1.0\times{10}_{50K}^{-3}$, $5.0\times{10}_{50K}^{-3}$, ${0.01}_{50K}$*,* ${0.05}_{50K}$*,* ${0.1}_{0.1M}$

**Key Points**

- This is the first time to build a graph-based encoder and transformer-based decoder model (named Phanto-IDP) to generate accurate protein backbones.
- The performance of Phanto-IDP is better than that of other methods on all 10 IDPs and four structured proteins.
- Phanto-IDP could also sample unseen conformations at an extremely low computational cost.

## Supplementary Material

phanto_idp_si_0920_bbad429

## Data Availability

The code for training Phanto-IDP and weights for generating conformation ensembles are available at https://github.com/HFChenLab/PhantoIDP. The trajectories involved in this article will be shared on reasonable request to the corresponding author.

## Author Biographies

**Junjie Zhu** is an undergraduate student studying bioinformatics at Shanghai Jiao Tong University. His research direction is molecular dynamics simulations, enhanced sampling and deep learning.

**Zhengxin Li** is a PhD student studying bioinformatics at Shanghai Jiao Tong University. His research direction is molecular dynamics simulations and biological force field.

**Haowei Tong** is an undergraduate who is major in bioinformatics at Shanghai Jiao Tong University. His research direction is t protein structure prediction via deep learning.

**Zhouyu Lu** is an undergraduate who is major in bioinformatics at Shanghai Jiao Tong University. His research direction is protein structure prediction via deep learning.

**Ningjie Zhang** is a master who is major in bioinformatics at Shanghai Jiao Tong University. Her research direction is protein conformation generation via deep learning.

**Ting Wei** is an assistant professor who is major in bioinformatics at Shanghai Jiao Tong University. Her research direction is protein design based on deep learning.

**Hai-Feng Chen** is a professor who is major in bioinformatics at Shanghai Jiao Tong University. His research direction is protein design based on deep learning and innovation drug design.
