# Supplementary material for: Phanto-IDP: compact model for precise intrinsically disordered protein backbone generation and enhanced sampling
Source: Brief Bioinform. 2023 Nov 28;25(1):bbad429. doi: 10.1093/bib/bbad429 (PMC10783862; doi:10.1093/bib/bbad429)
Supplement: phanto_idp_si_0920_bbad429 [file phanto_idp_si_0920_bbad429.docx]

# Supplementary Information

# Phanto-IDP: Compact Model for Precise Intrinsically Disordered Protein Backbone Generation Model and Enhanced Sampling

Junjie Zhu, Zhengxin Li, Haowei Tong, Zhouyu Lu, Ningjie Zhang, Ting Wei*, Hai-Feng Chen^*^

State Key Laboratory of Microbial metabolism, Joint International Research Laboratory of Metabolic & Developmental Sciences, Department of Bioinformatics and Biostatistics, National Experimental Teaching Center for Life Sciences and Biotechnology, School of Life Sciences and Biotechnology, Shanghai Jiao Tong University, Shanghai, 200240, China

***Corresponding Author**

**Hai-Feng Chen (Full Professor)**

State Key Laboratory of Microbial metabolism, Joint International Research Laboratory of Metabolic & Developmental Sciences, Department of Bioinformatics and Biostatistics, National Experimental Teaching Center for Life Sciences and Biotechnology, School of Life Sciences and Biotechnology, Shanghai Jiao Tong University, Shanghai, 200240, China

**Tel**: 86-21-34204073; **Fax**: 86-21-34204073; **Email**: [haifengchen@sjtu.edu.cn](mailto:haifengchen@sjtu.edu.cn); weitinging@sjtu.edu.cn

**Notes**

The authors declare that there is no conflict of interest.

**Table S1.** Comparison of Phanto-IDP against traditional methods on 10 systems involved in supplementary information.

| Protein | Method | Avg. RMSD, Å | JS Divergence of Rg | Speed |
| --- | --- | --- | --- | --- |
| Histain5 | AE | 7.038 | 1.792 | **10.31 s** |
|  | VAE | 5.412 | 0.834 | 15.65s |
|  | Phanto-IDP | **0.540** | **0.042** | 21.28s |
| Aβ40 | AE | 8.747 | 1.475 | **31.73 s** |
|  | VAE | 7.981 | 0.812 | 45.23s |
|  | Phanto-IDP | **0.596** | **0.048** | 32.94s |
| Aβ42 | AE | 9.267 | 1.835 | **29.45s** |
|  | VAE | 8.842 | 0.857 | 52.65s |
|  | Phanto-IDP | **1.086** | **0.052** | 33.07s |
| drkN SH3 domain | AE | 10.904 | 1.409 | 47.57s |
|  | VAE | 10.451 | 0.963 | 59.23s |
|  | Phanto-IDP | **0.693** | **0.079** | **35.32s** |
| ACTR | AE | 9.834 | 1.484 | 65.73s |
|  | VAE | 8.427 | 0.832 | 87.38s |
|  | Phanto-IDP | **0.516** | **0.072** | **36.26s** |
| R17 | AE | 11.983 | 1.321 | 107.22s |
|  | VAE | 12.179 | 0.945 | 159.84s |
|  | Phanto-IDP | **0.783** | **0.092** | **45.18s** |
| p15PAF | AE | 12.058 | 2.055 | 134,93s |
|  | VAE | 11.489 | 1.834 | 178.98s |
|  | Phanto-IDP | **0.987** | **0.110** | **52.54s** |
| CspTm | AE | 6.439 | 0.959 | 56.64s |
|  | VAE | 4.985 | 1.283 | 61.32s |
|  | Phanto-IDP | **0.187** | **0.025** | **35.68s** |
| Ubiquitin | AE | 8.432 | 0.916 | 78.54s |
|  | VAE | 6.231 | 1.578 | 91.03s |
|  | Phanto-IDP | **0.336** | **0.049** | **39.83s** |
| SPR17 | AE | 14.705 | 0.988 | 112.57s |
|  | VAE | 11.389 | 1.327 | 141.93s |
|  | Phanto-IDP | **0.683** | **0.083** | **48.34s** |

**
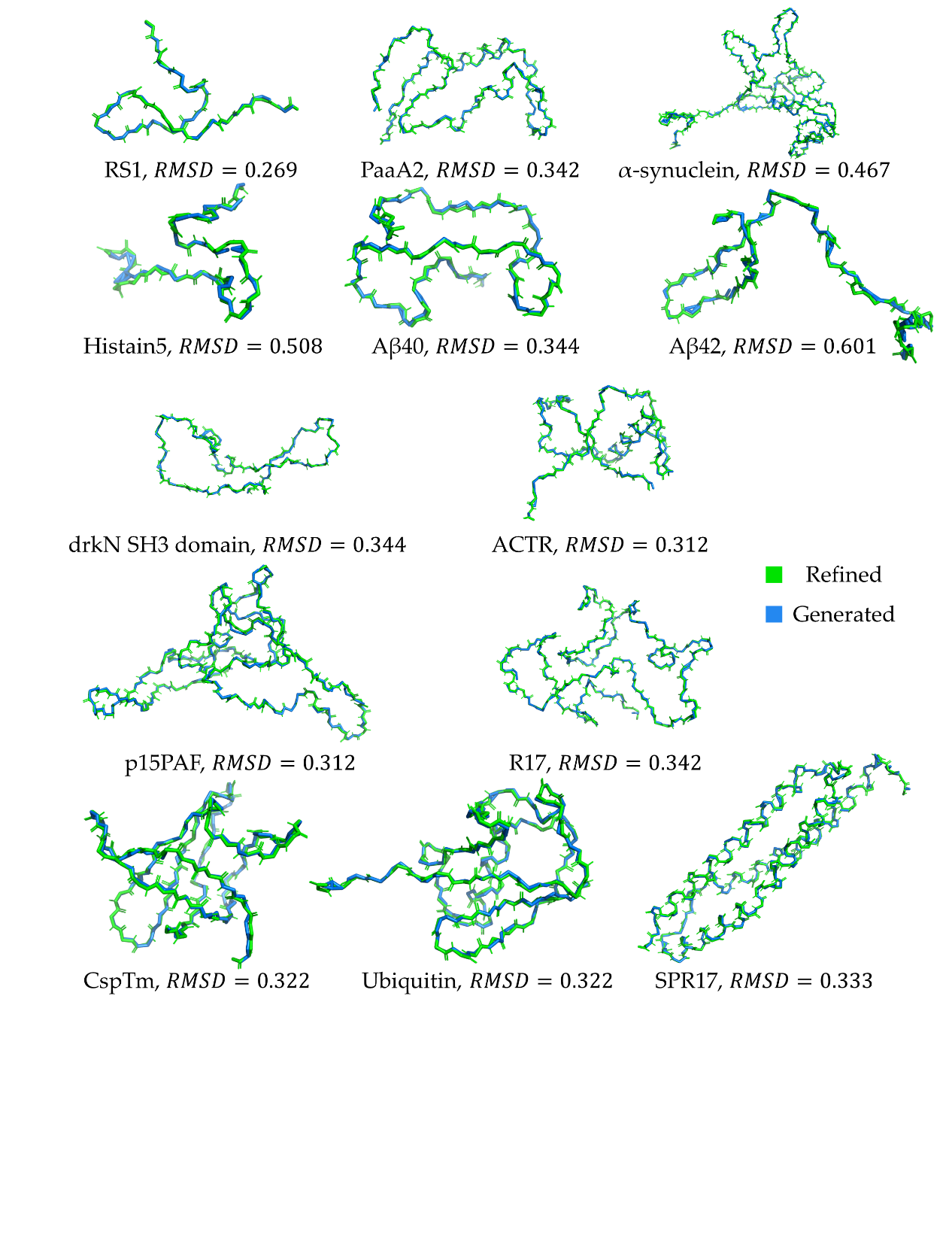
**

**Figure S1.** Comparison of the directly generated conformations to refined ones through RMSD (Å).

**
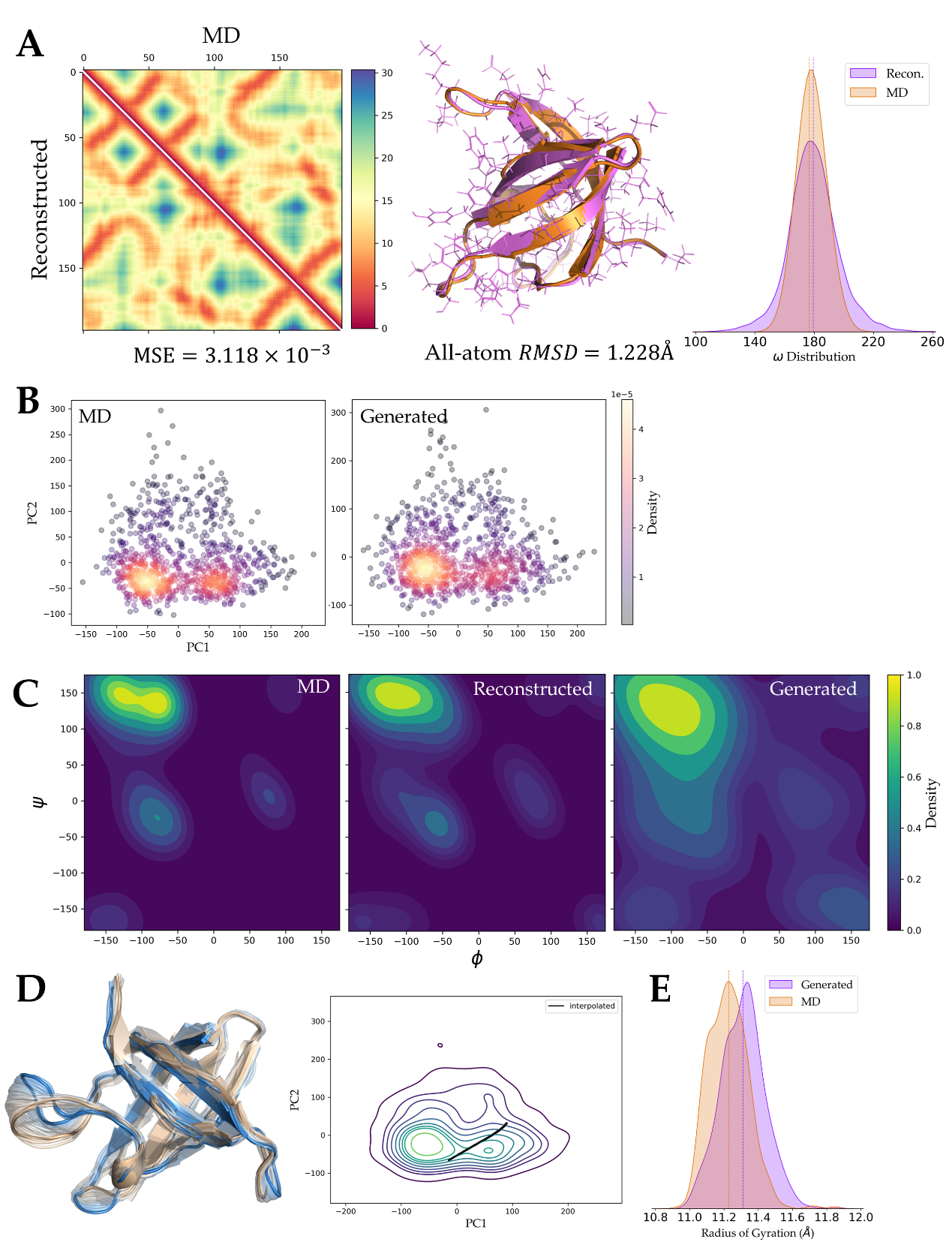
**

**Figure S2.** Evaluation on **CspTm**. (A) Comparison between reconstructed ensemble and MD trajectory. (B) Comparison between generated ensemble and MD trajectory. (C) Ramachandran plot of MD trajectory, reconstructed and generated ensemble. (D) Interpolation between selected conformations (that are extremely different). (E) Rg of generated ensemble compared to MD and experimental records.

**
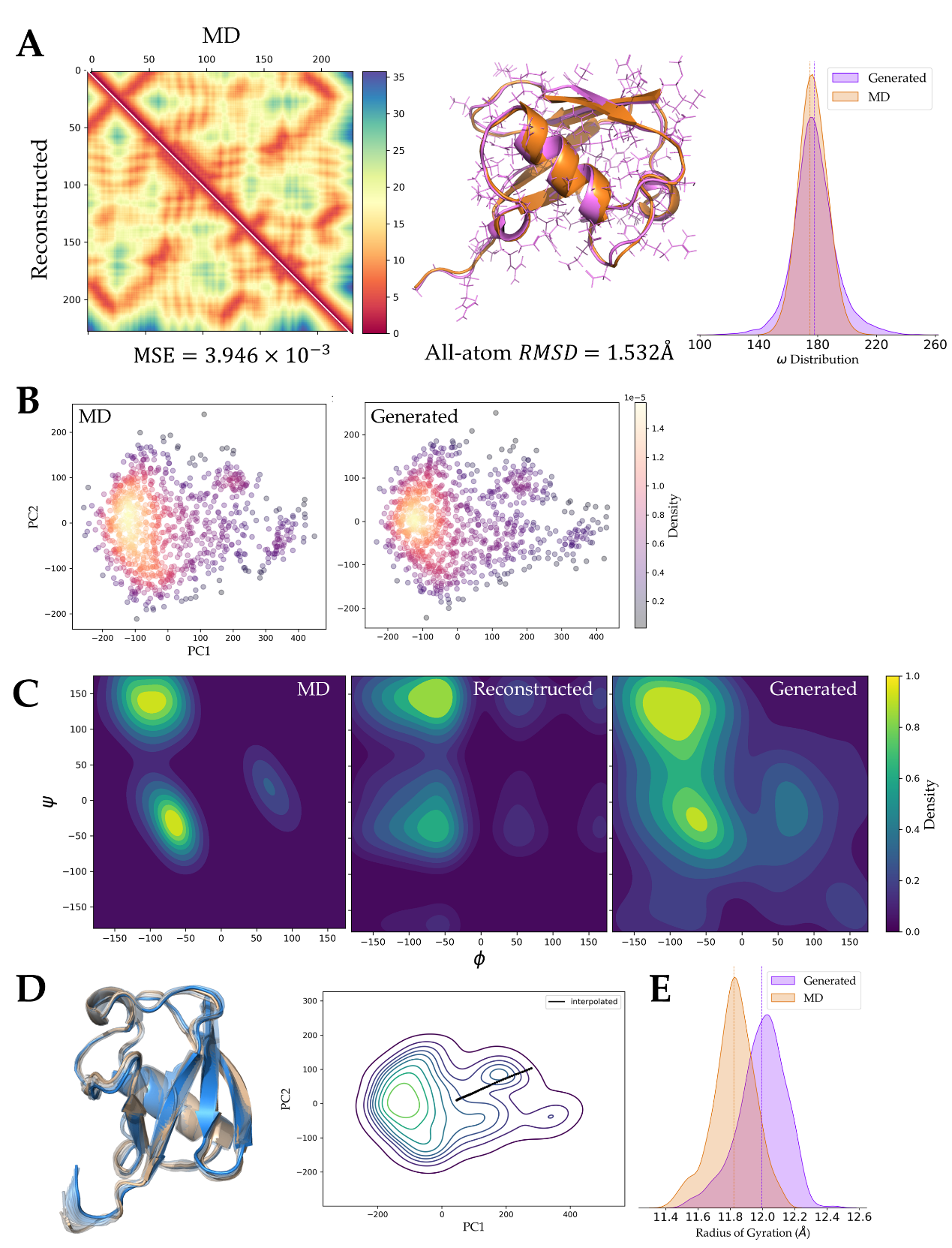
**

**Figure S3.** Evaluation on **Ubiquitin**. (A) Comparison between reconstructed ensemble and MD trajectory. (B) Comparison between generated ensemble and MD trajectory. (C) Ramachandran plot of MD trajectory, reconstructed and generated ensemble. (D) Interpolation between selected conformations (that are extremely different). (E) Rg of generated ensemble compared to MD and experimental records.

**
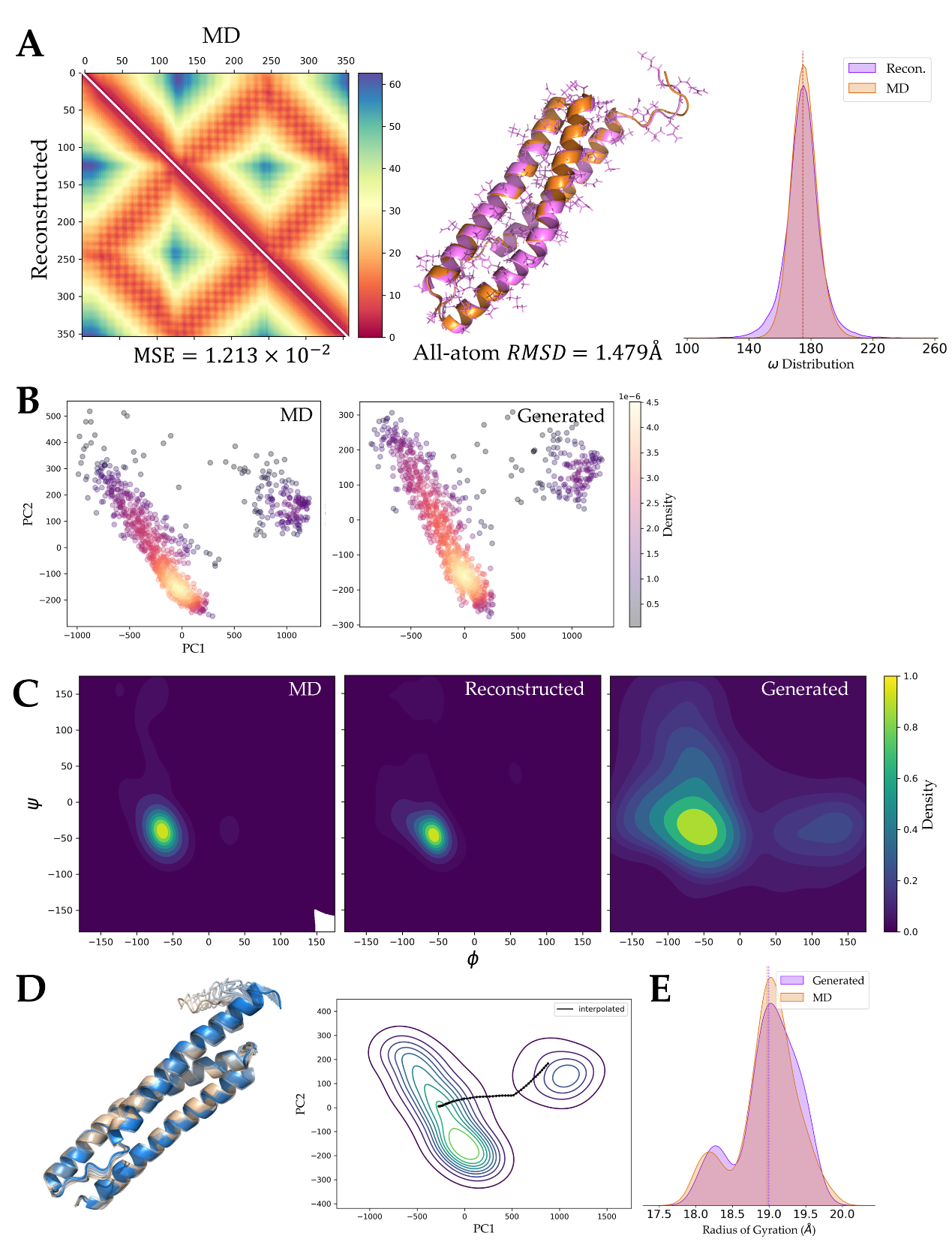
**

**Figure S4.** Evaluation on **SPR17**. (A) Comparison between reconstructed ensemble and MD trajectory. (B) Comparison between generated ensemble and MD trajectory. (C) Ramachandran plot of MD trajectory, reconstructed and generated ensemble. (D) Interpolation between selected conformations (that are extremely different). (E) Rg of generated ensemble compared to MD and experimental records.


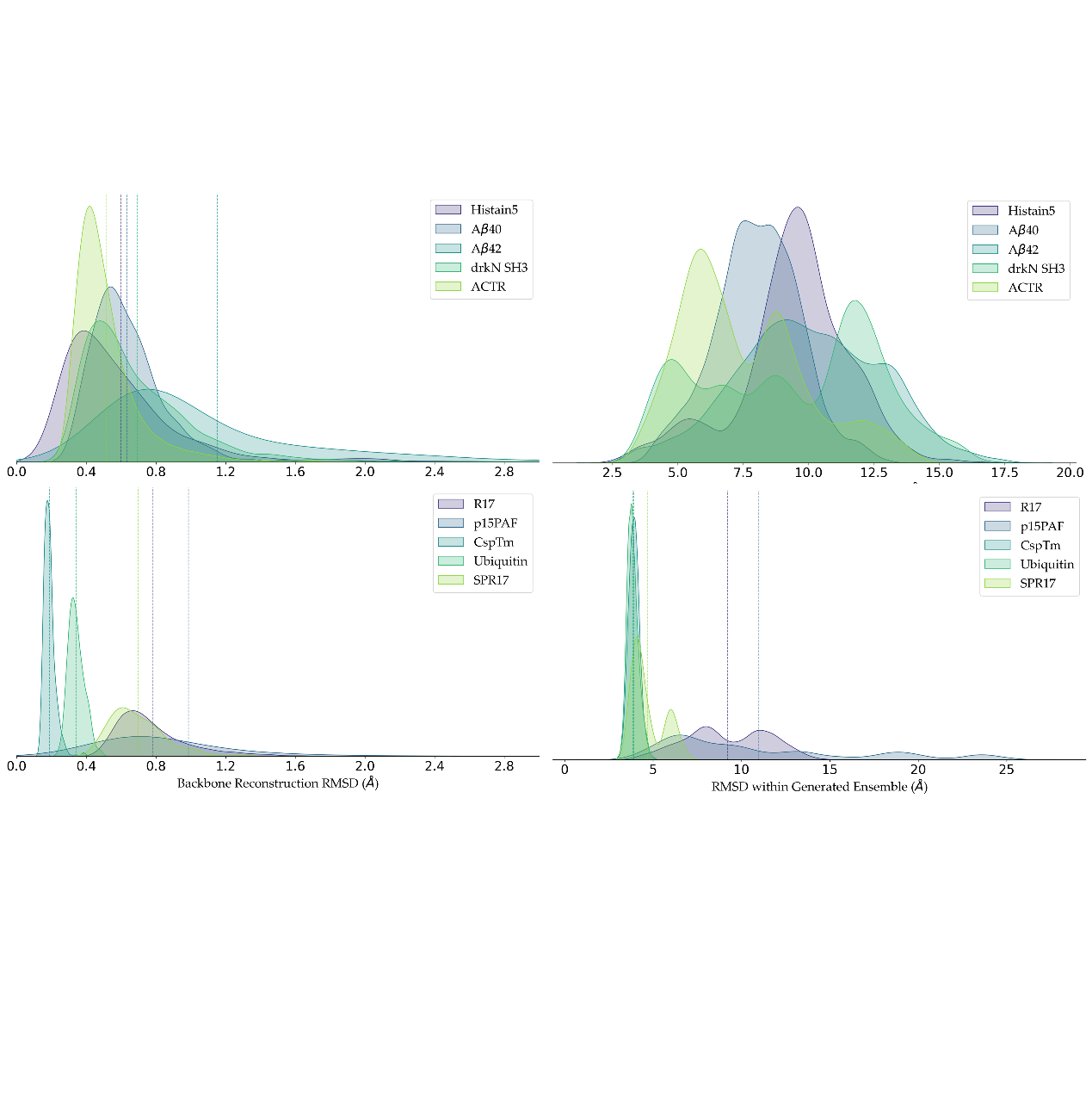


**Figure S5.** RMSD of reconstructed conformations against input ones (left) and RMSD within generated conformation ensembles (right) on 10 tested systems.

**
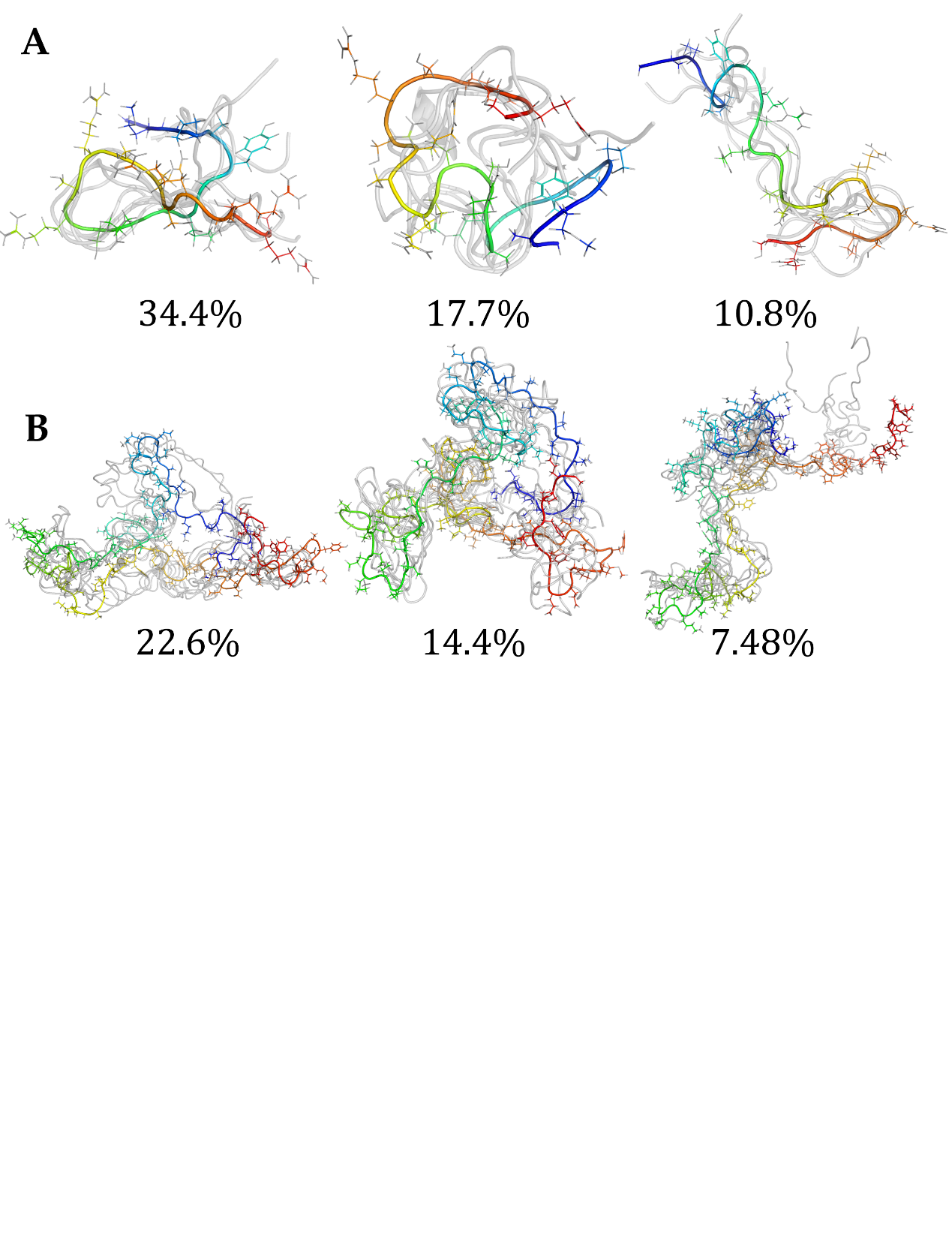
**

**Figure S6.** Result of clustering on generated conformation ensembles. (A) RS1. (B) α-synuclein.


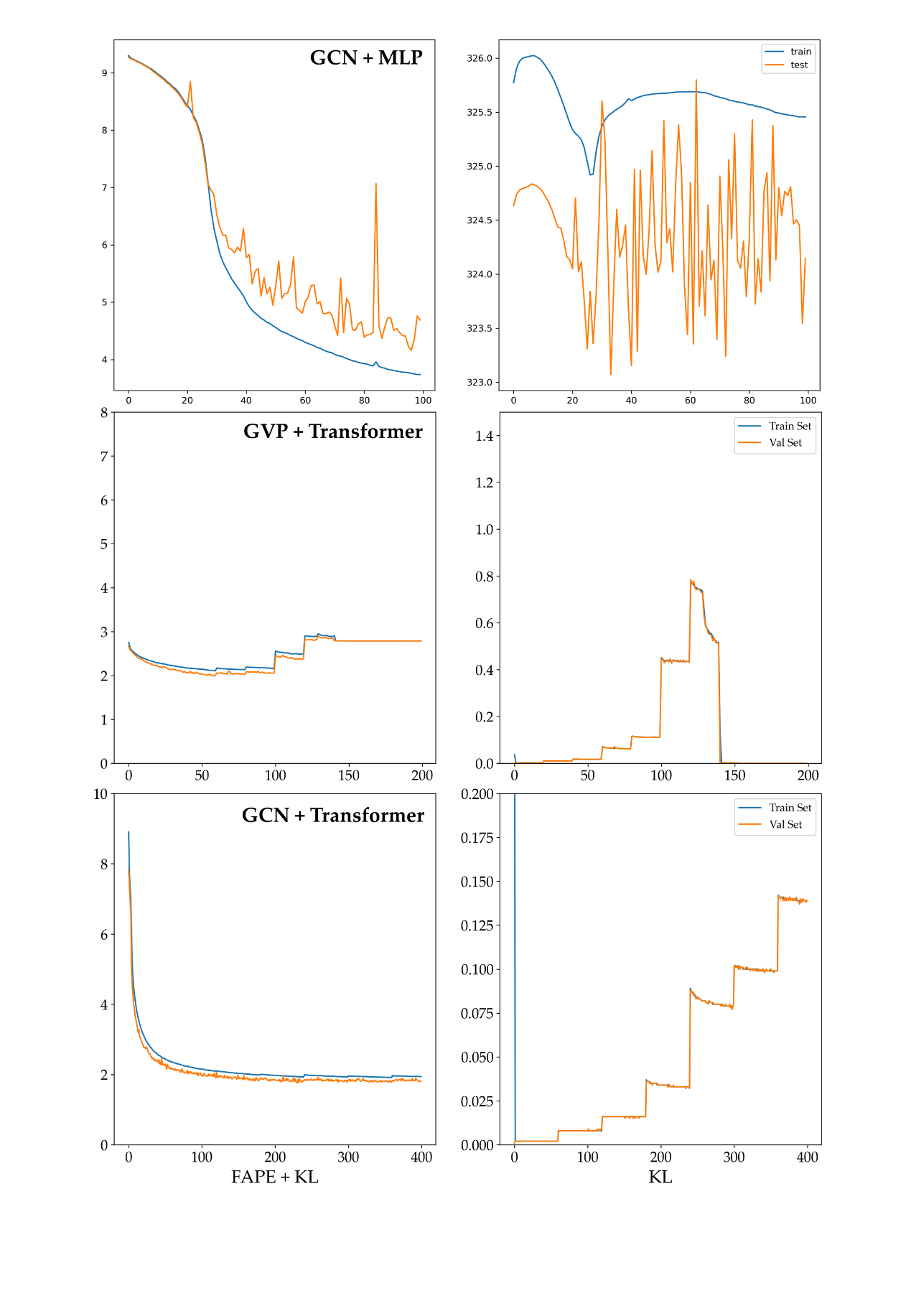


**Figure S7.** FAPE and KL loss on train set and validation set of PaaA2 on 3 different model structure settings.


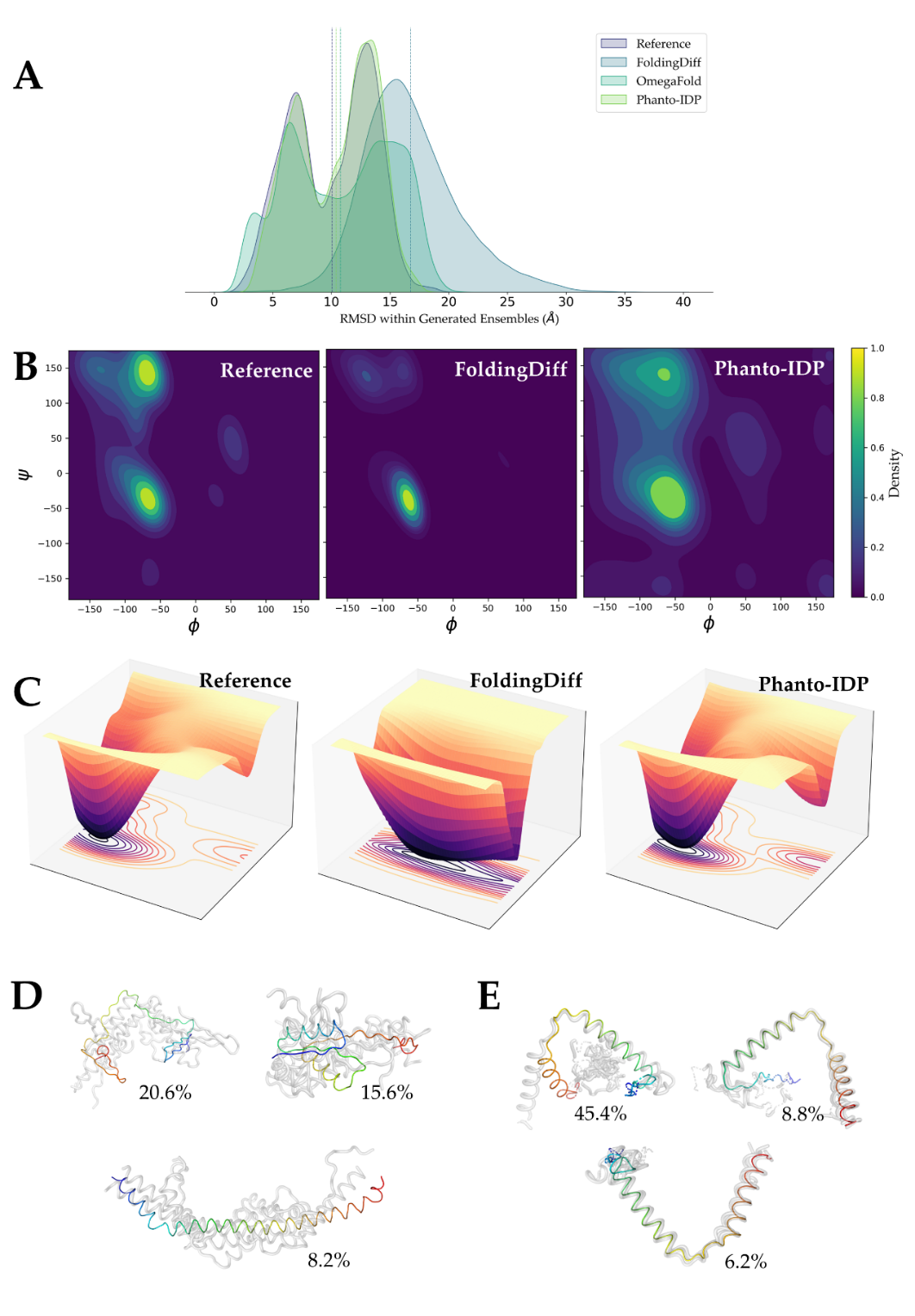


**Figure S8.** Comparison of Phanto-IDP against EigenFold and FoldingDiff on PaaA2. (A) RMSD within generated ensembles and MD trajectory. (B) Ramachandran plot of generated ensembles and MD trajectory. (C) PCA on backbone contact maps of generated ensembles and MD trajectory. (D) Clustering result of FoldingDiff. (E) Clustering result of EigenFold. Here reference refers to MD trajectory, and all plots involve 500 conformations.

**
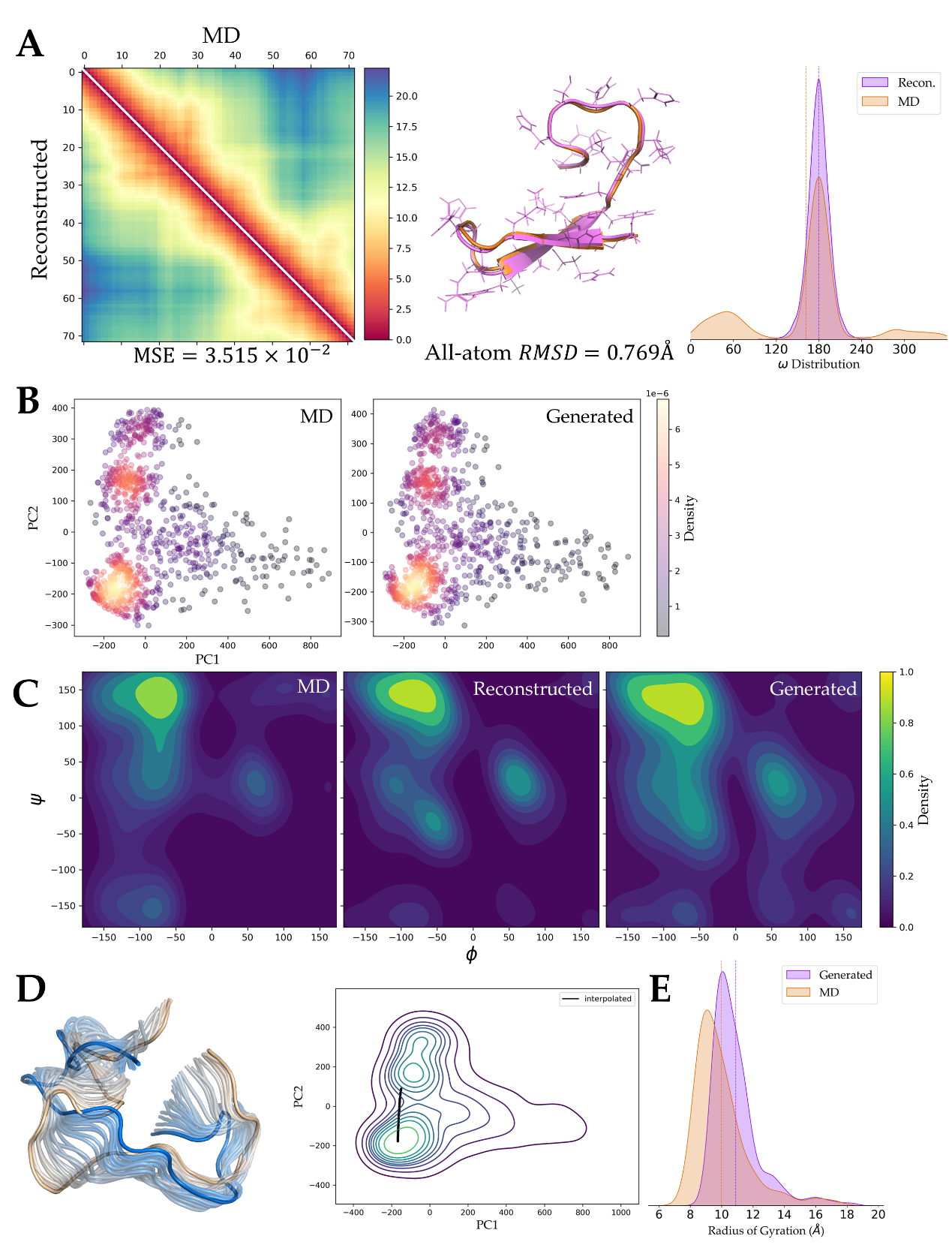
**

**Figure S9.** Evaluation on **Histain5**. (A) Comparison between reconstructed ensemble and MD trajectory. (B) Comparison between generated ensemble and MD trajectory. (C) Ramachandran plot of MD trajectory, reconstructed and generated ensemble. (D) Interpolation between selected conformations (that are extremely different). (E) Rg of generated ensemble compared to MD and experimental records.

**
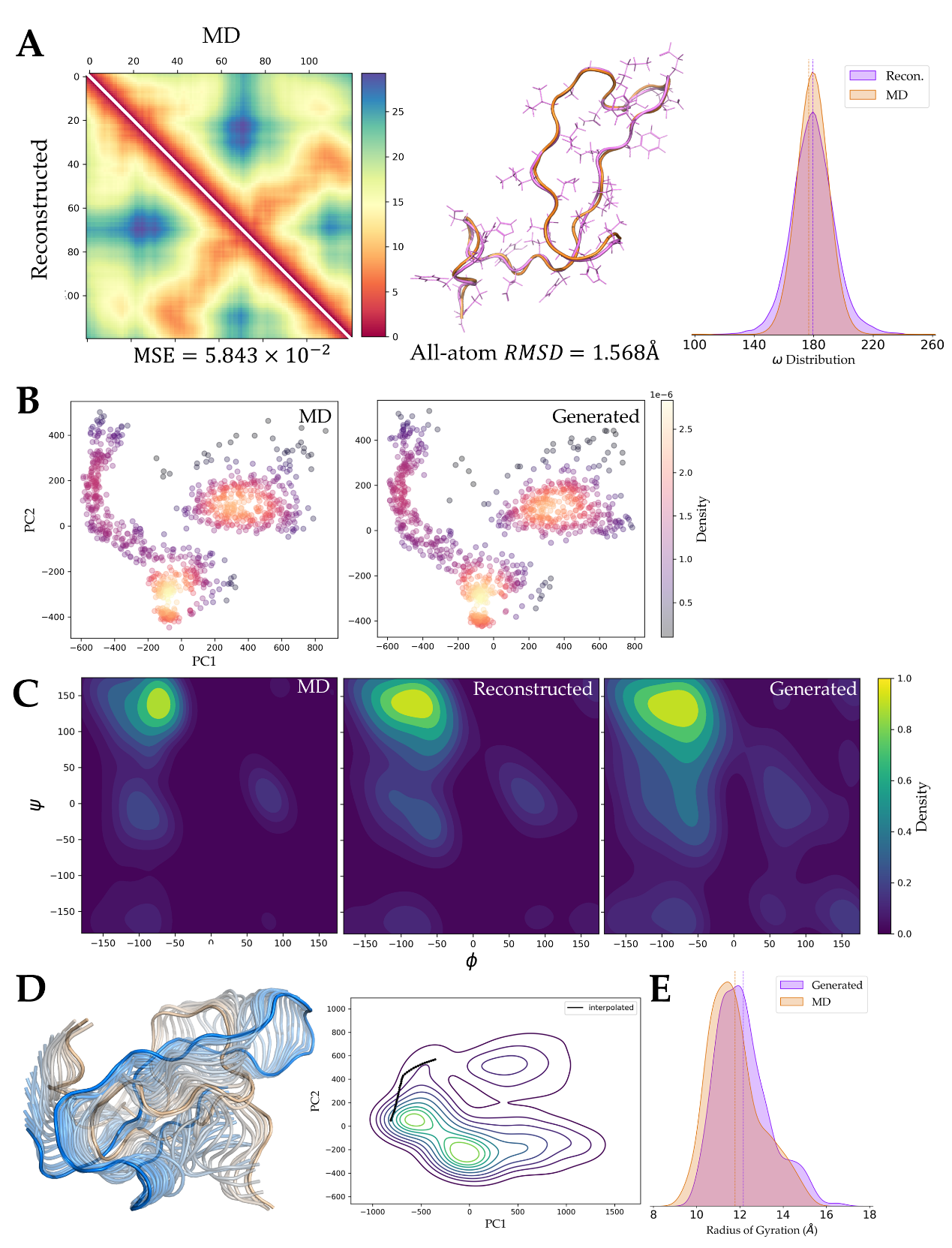
**

**Figure S10.** Evaluation on **Aβ40**. (A) Comparison between reconstructed ensemble and MD trajectory. (B) Comparison between generated ensemble and MD trajectory. (C) Ramachandran plot of MD trajectory, reconstructed and generated ensemble. (D) Interpolation between selected conformations (that are extremely different). (E) Rg of generated ensemble compared to MD and experimental records.

**
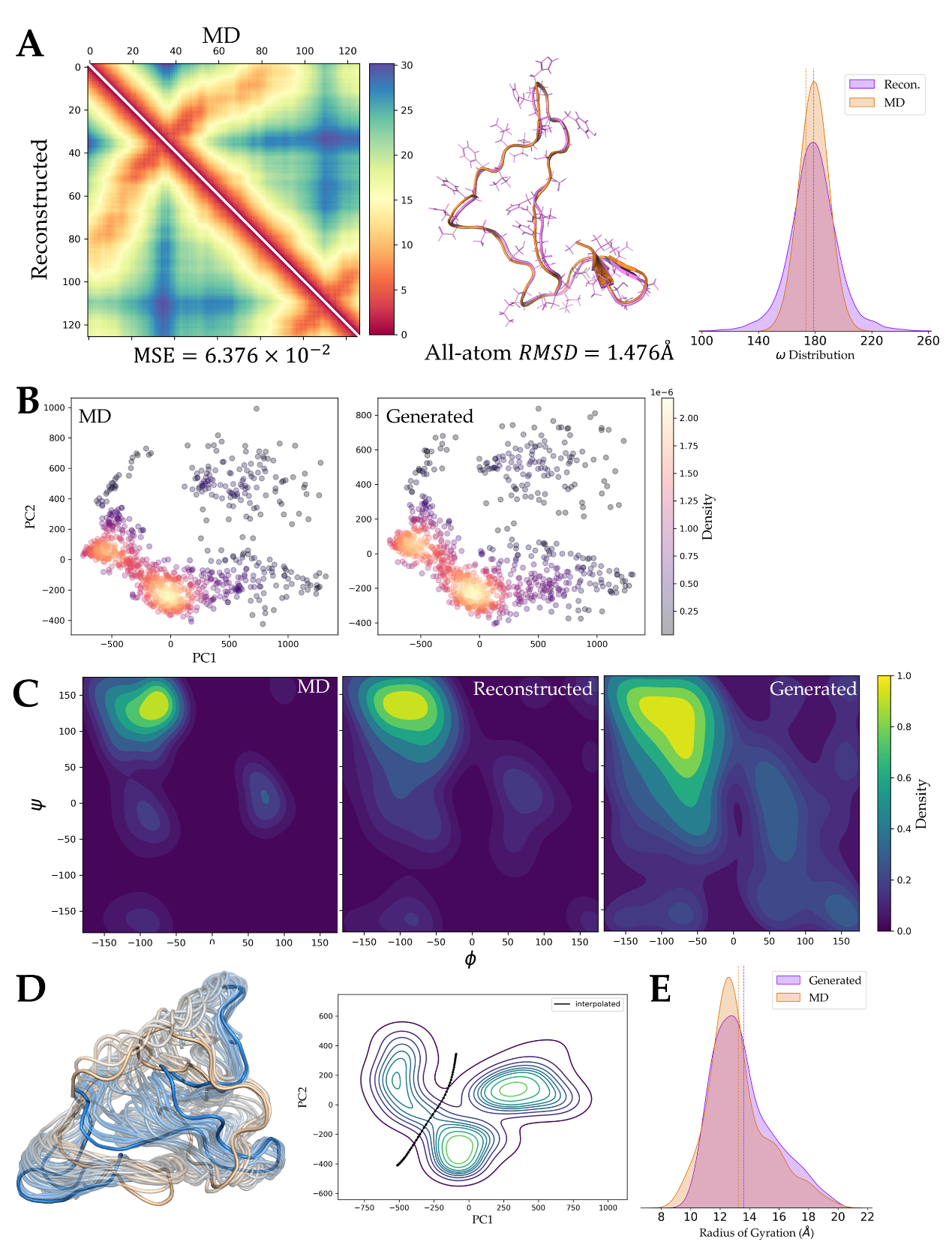
**

**Figure S11.** Evaluation on **Aβ42**. (A) Comparison between reconstructed ensemble and MD trajectory. (B) Comparison between generated ensemble and MD trajectory. (C) Ramachandran plot of MD trajectory, reconstructed and generated ensemble. (D) Interpolation between selected conformations (that are extremely different). (E) Rg of generated ensemble compared to MD and experimental records.

**
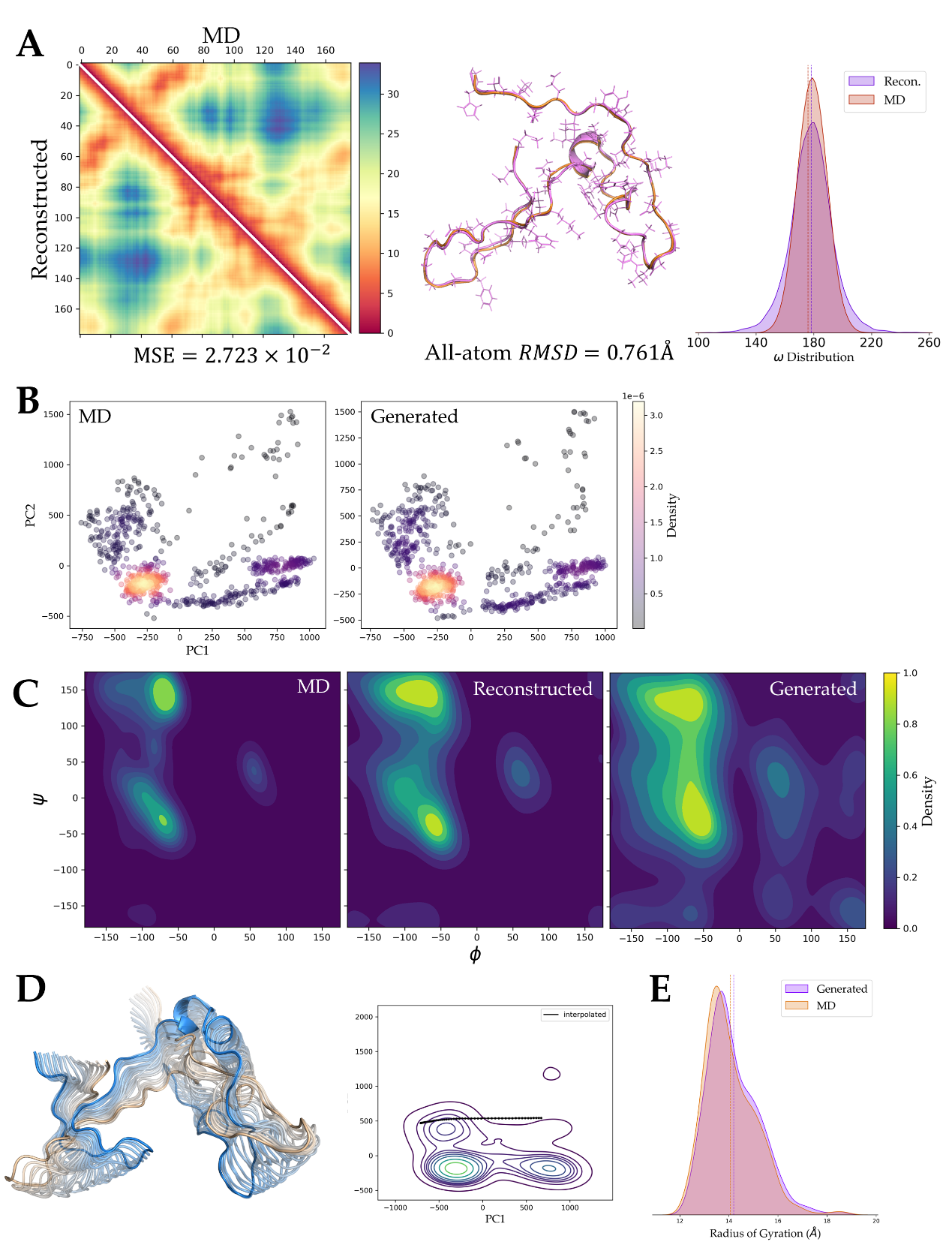
**

**Figure S12.** Evaluation on **drkN SH3 domain**. (A) Comparison between reconstructed ensemble and MD trajectory. (B) Comparison between generated ensemble and MD trajectory. (C) Ramachandran plot of MD trajectory, reconstructed and generated ensemble. (D) Interpolation between selected conformations (that are extremely different). (E) Rg of generated ensemble compared to MD and experimental records.

**
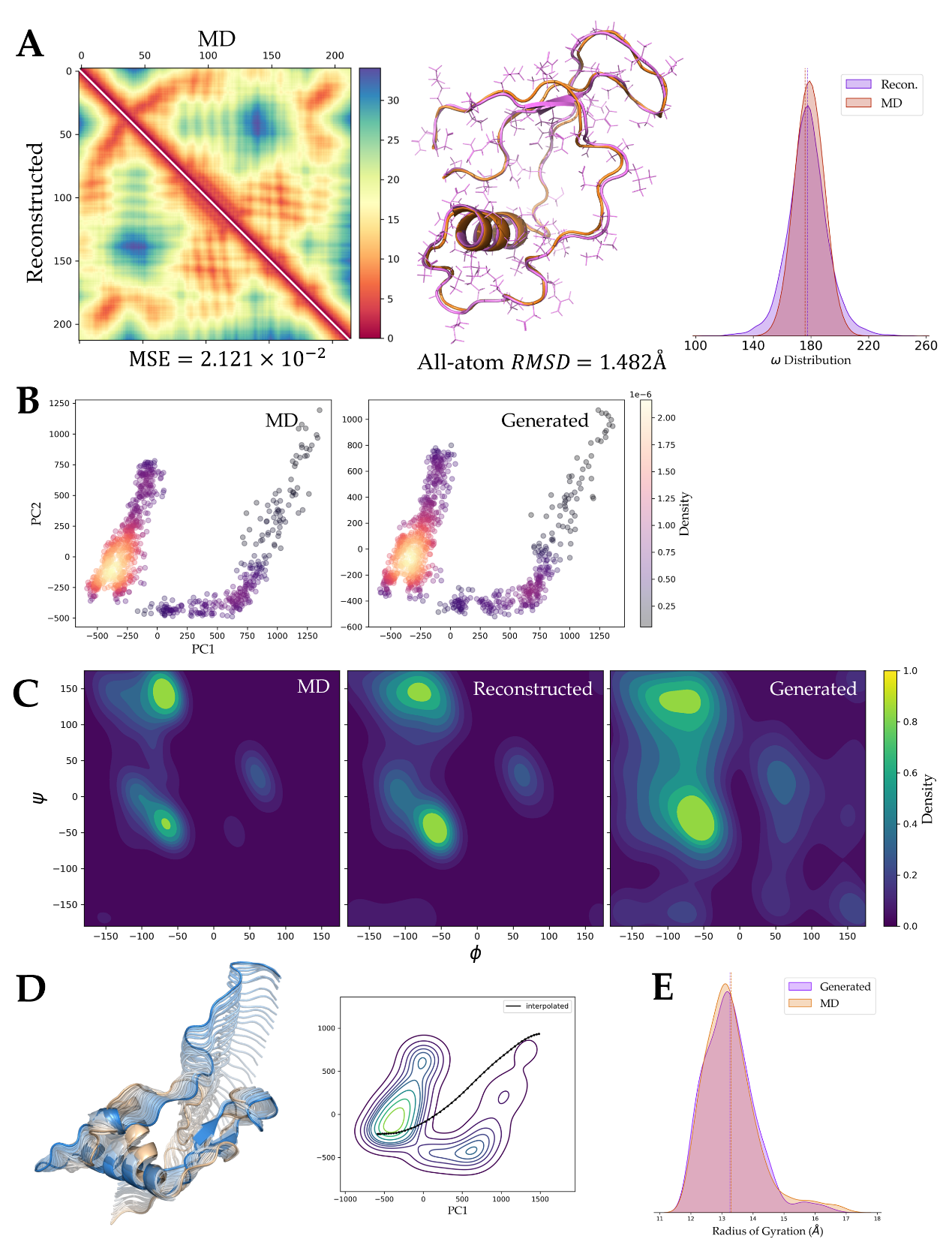
**

**Figure S13.** Evaluation on **ACTR**. (A) Comparison between reconstructed ensemble and MD trajectory. (B) Comparison between generated ensemble and MD trajectory. (C) Ramachandran plot of MD trajectory, reconstructed and generated ensemble. (D) Interpolation between selected conformations (that are extremely different). (E) Rg of generated ensemble compared to MD and experimental records.

**
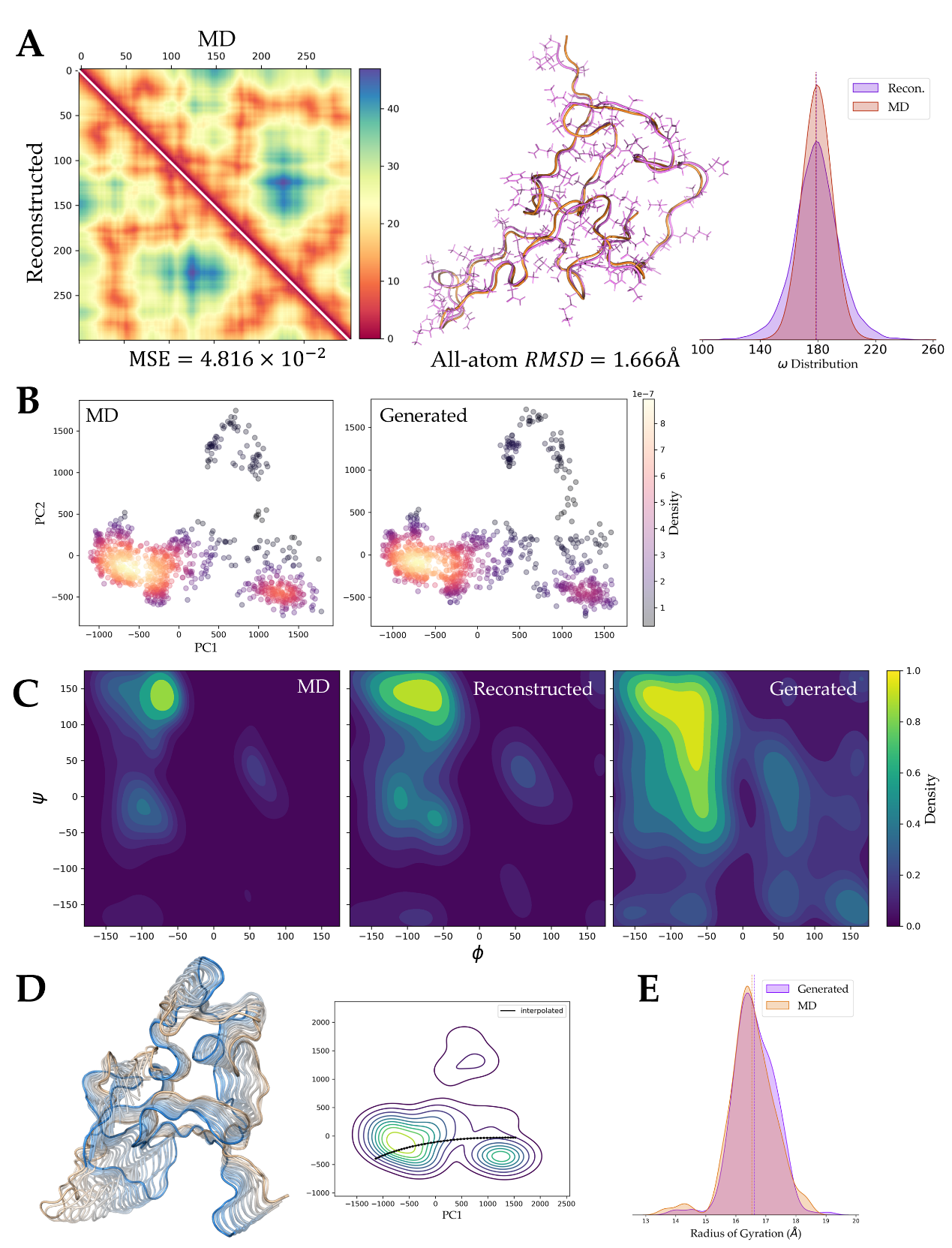
**

**Figure S14.** Evaluation on **R17**. (A) Comparison between reconstructed ensemble and MD trajectory. (B) Comparison between generated ensemble and MD trajectory. (C) Ramachandran plot of MD trajectory, reconstructed and generated ensemble. (D) Interpolation between selected conformations (that are extremely different). (E) Rg of generated ensemble compared to MD and experimental records.

**
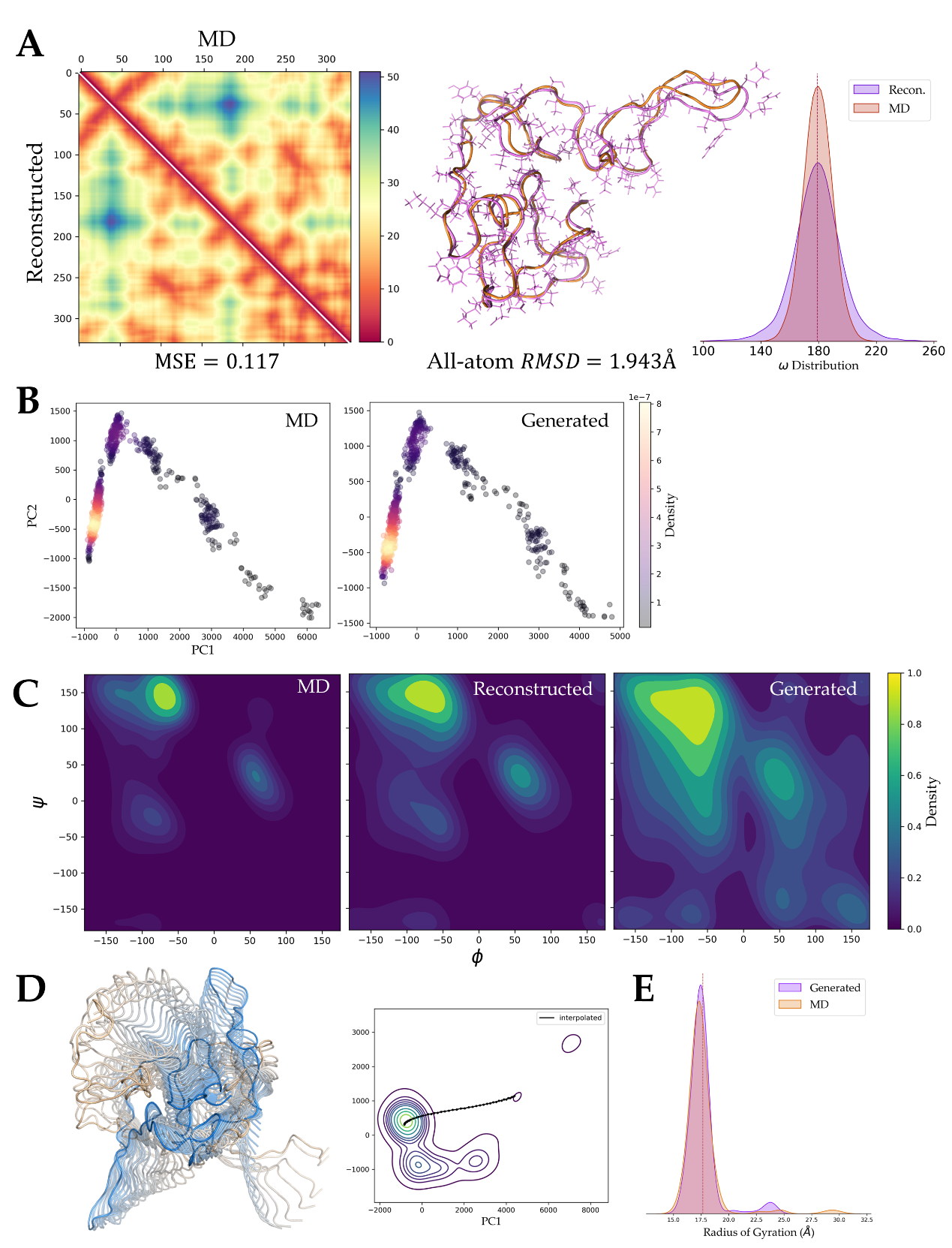
**

**Figure S15.** Evaluation on **p15PAF**. (A) Comparison between reconstructed ensemble and MD trajectory. (B) Comparison between generated ensemble and MD trajectory. (C) Ramachandran plot of MD trajectory, reconstructed and generated ensemble. (D) Interpolation between selected conformations (that are extremely different). (E) Rg of generated ensemble compared to MD and experimental records.


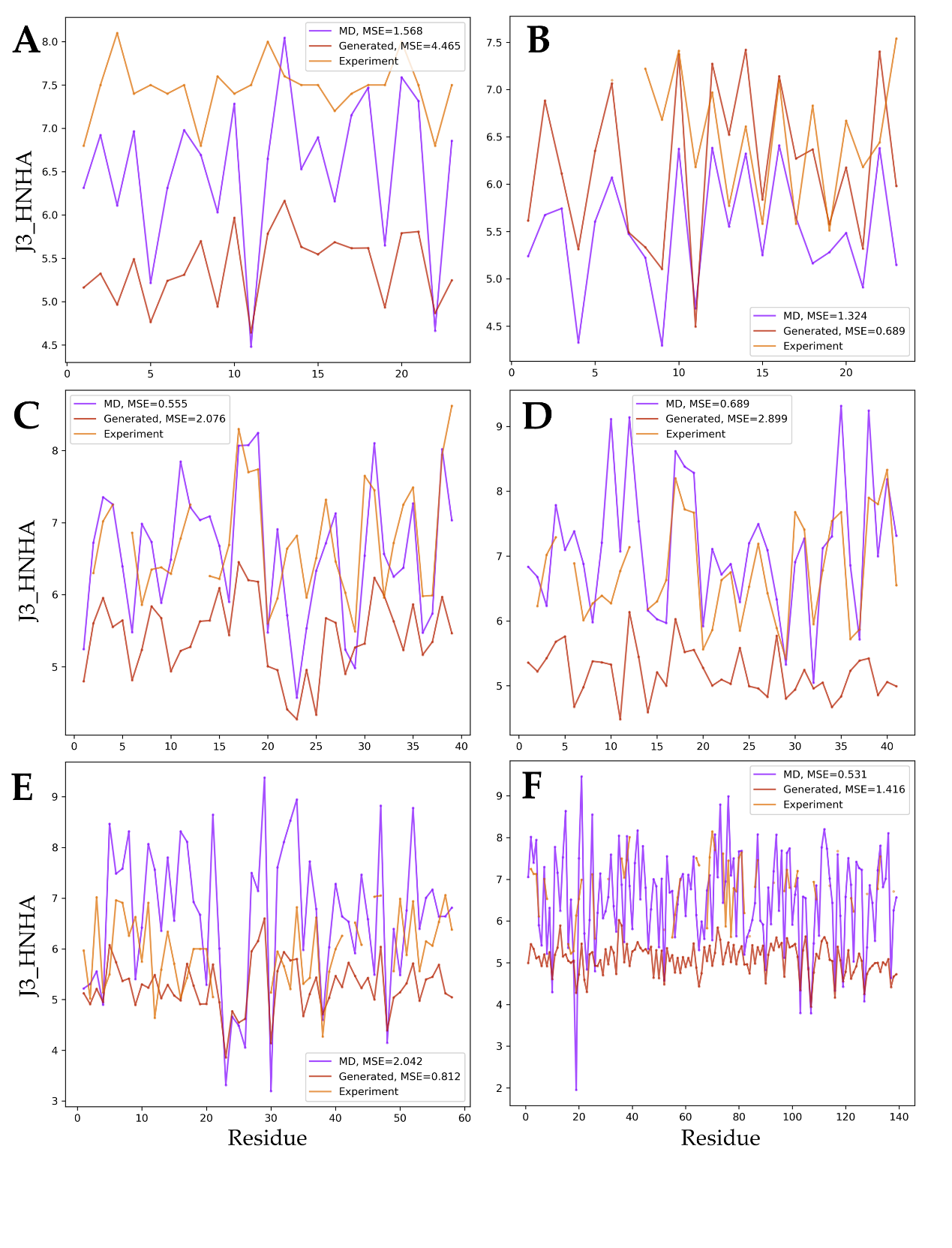


**Figure S16.** J-coupling of generated ensembles, MD trajectories and corresponding experimental values. (A) Histain5. (B) RS1. (C) Aβ40. (D) Aβ42. (E) drkN SH3 domain. (F) α-synuclein.


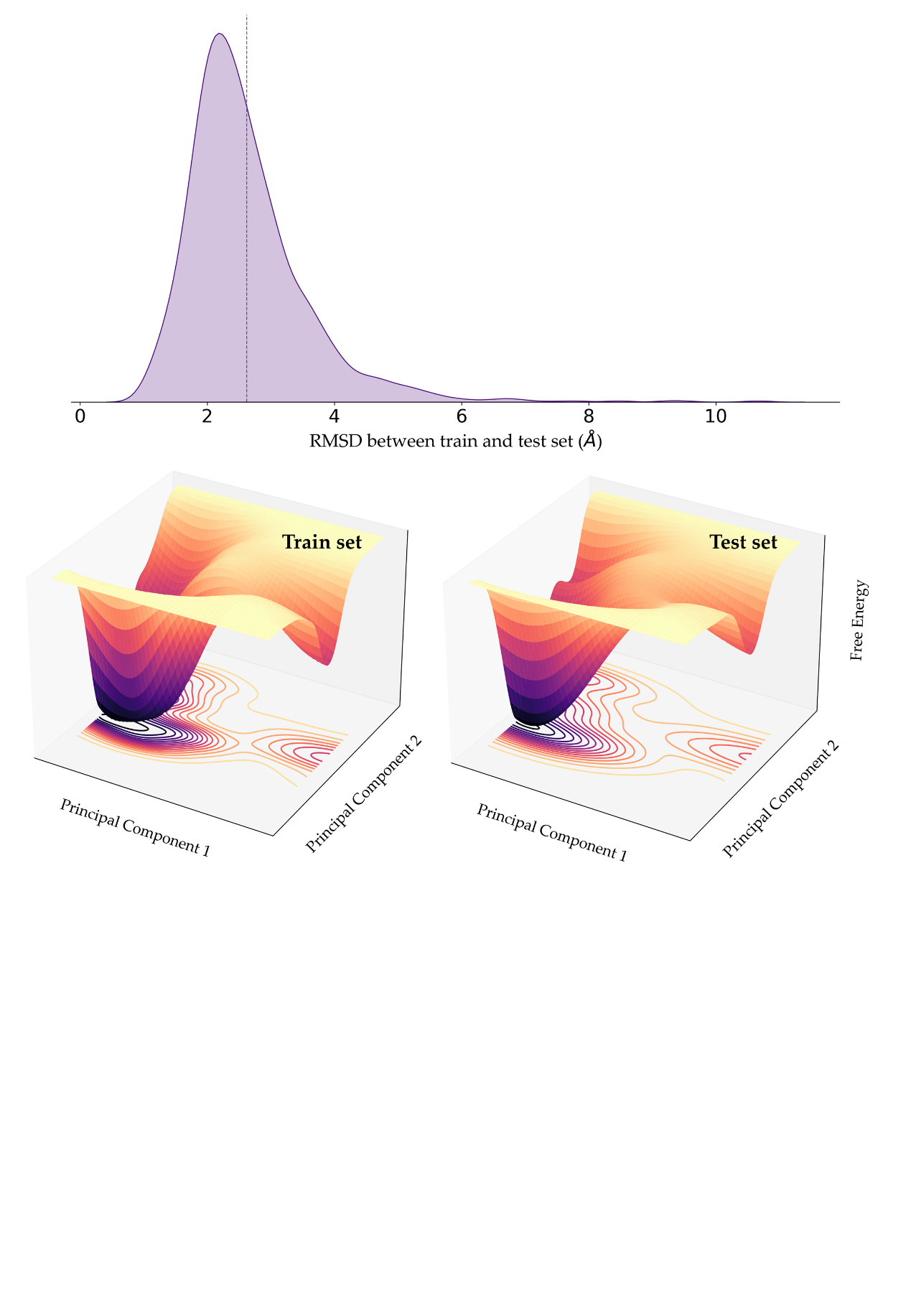


**Figure S17.** Comparison between train set and test set on PaaA2.
